# KIN-1 Function in *Neurospora crassa* Revealed by Constitutive *clr-2* Expression and Cross-Species Complementation

**DOI:** 10.3390/jof12100762

**Published:** 2026-10-10

**Authors:** Haowen Sun, Huizhen Chen, Pengchao Wei, Chanjuan Guo, Zheng Lin, Lei Guo, Liuyan Guo, Wenjing Ning, Jianzhong Huang, Lina Qin, Xianzhang Jiang

**Affiliations:** National and Local Joint Engineering Research Center of Industrial Microbiology and Fermentation Technology, College of Life Sciences, Fujian Normal University, Fuzhou 350100, China; sunhaowen96@163.com (H.S.); huizhenchen20241@163.com (H.C.); 13171068251@163.com (P.W.); gcjwishpos@163.com (C.G.); 18950729485@163.com (Z.L.); 15805033031@163.com (L.G.); 15377928925@163.com (L.G.); ningwenjing2003@163.com (W.N.); hjz@fjnu.edu.cn (J.H.)

**Keywords:** *Neurospora crassa*, kinesin-1, *clr-2*, cellulase, polarized growth, carbon source, heterologous complementation

## Abstract

Conventional kinesin contributes to fungal polarized growth, but its role in cellulase production remains unclear. We examined the effects of *kin-1* deletion, constitutive *clr-2* expression, and complementation with the native gene or a *Trichoderma reesei* gene in *Neurospora crassa*. Deletion of *kin-1* produced compact colonies, increased branching in the hyphae examined, and reduced extension rates without reducing biomass in sucrose cultures. Under Avicel conditions, the deletion strain showed reduced extracellular protein accumulation and lower *cbh-1* and *clr-2* transcript levels. Introduction of a *clr-2* expression cassette into the deletion background increased the transcript levels of both genes relative to *Δkin-1* and increased extracellular protein and cellulase activities under both Avicel and sucrose conditions. At a fixed total protein loading of 0.1 mg per saccharification reaction, performance varied with the carbon source used for enzyme production and the straw substrate. Heterologous complementation alleviated the growth defects. On day 7 of Avicel cultivation, extracellular protein normalized to intracellular protein was lower in the deletion strain and was restored to near-WT levels in both complemented strains. These results link KIN-1-dependent morphogenesis to the production of cellulolytic products and show that constitutive *clr-2* expression can alleviate some phenotypes caused by *kin-1* deletion. The extent to which the tested *T. reesei* gene substitutes for the native gene depends on the phenotype. This study does not establish direct cellulase transport by KIN-1, a change in secretion rate per unit biomass, or complete restoration of secretion.

## 1. Introduction

Fungi degrade lignocellulose by producing extracellular enzymes and secreting them into the surrounding substrate. *Neurospora crassa*, with its well-established genetic tools, is an important model for studying these processes. Systems-level studies have shown that growth on plant biomass is accompanied by changes in gene expression and the secreted proteome [1,2]. In the industrial enzyme-producing fungus *Trichoderma reesei*, strain improvement addresses both regulatory and physiological factors that affect cellulase production [3]. Understanding how growth-related mechanisms influence enzyme production can help explain differences in productivity among strains with distinct morphologies.

In *N. crassa*, cellulase induction depends on the identity and availability of extracellular carbon sources. Cellodextrins released during cellulose degradation induce cellulolytic enzymes [4]. The functions of the cellodextrin transporters CDT-1 and CDT-2 suggest that, in addition to substrate uptake, they may participate in nutrient sensing [5]. CDT-2 also contributes to cellulose and hemicellulose utilization [6]. The cellulase regulatory protein CLP1 provides a further link between cellodextrin metabolism and enzyme induction [7]. These findings place carbon-source sensing upstream of enzyme production; reduced extracellular enzyme levels therefore cannot be attributed solely to defective protein transport.

Transcriptional regulation is another important determinant of cellulolytic enzyme production. CRE-1 mediates carbon catabolite repression, whereas CLR-1 and CLR-2 are essential regulators of the cellulose response [8,9]. Constitutive *clr-2* expression can bypass the usual requirement for cellulose induction in parts of this response [10]. Direct target analyses have defined the regulatory networks controlled by CLR-1 and CLR-2 [11]. VIB1 and the CLR-3/OS signaling system connect cellulolytic regulation to nutrient-responsive pathways [12,13], although transcriptomic responses to different carbon sources are not governed by a single regulator [14]. Constitutive *clr-2* expression can thus be used to test whether activation of downstream cellulolytic gene expression alleviates a phenotypic defect. This experiment alone, however, cannot identify the molecular cause of that defect.

Extracellular protein and enzyme production also depend on the organization of hyphal growth and membrane trafficking. Filamentous fungi grow through localized cell-wall expansion involving the cytoskeleton, secretory organelles, and the Spitzenkörper [15,16,17]. Protein secretion associated with hyphal tips has been observed in *Aspergillus niger* [18]. In *N. crassa*, the exocyst participates in delivery at the Spitzenkörper and apical plasma membrane [19], while live-cell studies in *Aspergillus nidulans* have linked late-Golgi maturation to the formation of post-Golgi secretory carriers [20]. Changes in the number or organization of growing tips may therefore affect secretory physiology, but increased branching does not by itself imply increased enzyme production.

Conventional kinesin may connect morphogenesis with extracellular protein production. Biochemical studies of *N. crassa* kinesin [21] and loss-of-function studies have implicated it in polarized secretion, morphogenesis, and hyphal tip organization [22,23]. Comparative studies have shown that kinesin families are diverse across filamentous ascomycetes [24]. In other fungi, kinesin-dependent transport delivers Rab11 secretory vesicles [25] and cell-wall synthases [26,27,28]. These findings do not establish that cellulases are direct KIN-1 cargoes or that KIN-1 directly regulates cellulase gene transcription. Testing whether transcriptional activation alleviates a motor-associated cellulolytic phenotype is distinct from identifying the cargoes transported by that motor.

Morphological engineering studies further show why growth morphology and enzyme production should be evaluated together rather than treated as interchangeable measures. Deletion of *gul-1* in *N. crassa* alters morphology and increases cellulase production [29], whereas hyperbranching caused by *racA* deletion in *A. niger* does not necessarily increase secretion [30]. Fungal morphology can affect growth, broth properties, and product formation, with effects that depend on the species and culture conditions [31]. Studies of kinesin mutants therefore need to measure hyphal extension, biomass, extracellular protein, and catalytic activity together.

We used three sets of genetic comparisons to investigate the effects of *kin-1* deletion in *N. crassa*. First, we compared growth, morphology, biomass, and extracellular protein accumulation in WT, *Δkin-1*, and the strain complemented with the native gene, *Ckin-1*, and compared selected cellulolytic transcripts between WT and *Δkin-1*. We then introduced a constitutive *clr-2* expression cassette into the deletion background and examined extracellular protein, cellulase activities, and straw saccharification under Avicel and sucrose conditions. Finally, complementation with the *T. reesei kin-1* gene tested whether the donor gene could substitute for the native gene in growth and enzyme-related functions. This design distinguishes transcriptional compensation from gene complementation and evaluates functional conservation for each measured phenotype.

## 2. Materials and Methods

### 2.1. Strains and Genetic Design

The *N. crassa* wild-type strain was FGSC 2489 (OR74A background), in which the *kin-1* locus corresponds to NCU09730 (NCBI Gene ID 3880594). The experimental strains were *Δkin-1*, *Ckin-1*, *WT::Mclr-2*, *Δkin-1::Mclr-2*, and *Δkin-1::kinesin* (Table 1). *Escherichia coli* DH5α was used for plasmid propagation. The *kin-1* open reading frame was replaced with the hygromycin-resistance marker *hph* by homologous recombination. *Ckin-1* was constructed using the native *kin-1* genomic fragment, including its flanking regulatory sequences, and introduced into the deletion background by crossing. *WT::Mclr-2* carries a *clr-2* expression cassette driven by the *ccg-1* promoter at the *his-3* locus; crossing this strain with *Δkin-1* yielded *Δkin-1::Mclr-2*. The heterologous kinesin gene was obtained from *T. reesei* QM6a (locus TRIREDRAFT_55060; NCBI Gene ID 18485786; reference protein XP_006962313.1) and introduced into *Δkin-1* to generate *Δkin-1::kinesin*. The database accession numbers identify the donor locus.

Strain construction and PCR screening are described in Supplementary Methods S1–S4 and Figures S1–S4. The PCR screening records shown include 7 deletion candidates, 5 native-complementation candidates, 5 *Δkin-1::Mclr-2* candidates, and the heterologous-complementation samples in Figure S3. These numbers refer only to the candidates documented in the figures, not to the total number of colonies screened. Additional colony phenotypes and extracellular protein profiles are shown in Figures S5 and S6, respectively. Experimental sequencing reads and their coverage are presented in Figure S7 and Supplementary Data S1. Hyphal morphology of independent isolates is shown in Figure S8. Oligonucleotide sequences are listed in Table S1. *Ckin-1* denotes the strain complemented with the native gene. *Mclr-2* denotes the P*ccg-1*-driven *clr-2* expression cassette and does not indicate a mutation in the CLR-2 coding sequence.

### 2.2. Culture Conditions and Growth Phenotyping

Strains were maintained on Vogel’s medium containing 2% (*w*/*v*) sucrose. Colonies were imaged after 48 h of incubation at 28 °C in the dark, and hyphal morphology was examined after 12 h. Aerial hyphal growth was assessed qualitatively after approximately 7 d of slant culture. Linear extension was measured every 24 h in race tubes containing 15 mL of solid Vogel’s medium with 2% sucrose at 28 °C and expressed as cm day^−1^. Colony radial growth in the heterologous-complementation experiments is reported separately in mm day^−1^ and was not pooled with race-tube extension data.

Dry biomass was measured in 24-well plates containing 3 mL of Vogel’s medium with 2% sucrose per well. Each well was inoculated with 10^6^ conidia and incubated at 28 °C and 580 rpm. Samples were collected every 24 h up to 168 h. Mycelia were separated through four layers of gauze and dried at 65 °C to constant weight. The same culture and measurement procedures were used in the complementation experiments.

Selected main hyphal axes were traced in the micrographs shown, and associated branch points, including higher-order branches, were counted. The denominator for branch density was the traced main-axis length, not the total length of all descendant branches. Axis segments within the same image field or connected network were treated as image observations, not independent culture replicates. The main figures retain the values and annotations in the supplied branch-density plots; the corresponding image records and numerical checks are provided in Supplementary Data S3.

For protein and enzyme production, 250 mL Erlenmeyer flasks containing 50 mL of Vogel’s medium with 2% (*w*/*v*) Avicel or sucrose were inoculated at a final concentration of 10^6^ conidia mL^−1^ and incubated at 28 °C and 220 rpm for 7 d. Samples were collected every 24 h and centrifuged at 13,000 rpm for 10 min; the supernatants were stored at 4 °C until analysis. Protein comparisons involving *Δkin-1* and the native-complemented strain, and protein and enzyme activity comparisons involving the heterologous-complemented strain, were performed under Avicel conditions. *Mclr-2* strains were examined under both Avicel and sucrose conditions. Complete medium formulations are provided in Supplementary Method S5.

### 2.3. Extracellular Protein Measurement and SDS–PAGE

Extracellular protein concentration was measured by the Bradford assay using bovine serum albumin as the standard [32]. Diluted culture supernatant or standard (5 μL) was mixed with 250 μL of Bradford reagent and incubated at room temperature for 5 min. Absorbance at 595 nm was measured with a SpectraMax i3X microplate reader (Molecular Devices, LLC, San Jose, CA, USA). Protein concentration was calculated from the standard curve and expressed as protein mass per mL of culture supernatant (mg mL^−1^). Samples from the complementation series collected on day 7 of Avicel cultivation were also normalized to intracellular protein as described below.

For SDS–PAGE, culture supernatants collected at 168 h were mixed with 5× loading buffer at a ratio of 4:1, heated in a boiling-water bath for 10 min, and centrifuged at 13,000 rpm for 10 min. Equal sample volumes (20 μL) were loaded, and electrophoresis was performed initially at 80 V and then at 120 V. Gels were stained with Coomassie Brilliant Blue R-250 for at least 3 h, destained, and imaged. This loading scheme compares equal sample volumes, not equal protein amounts. Bands were not assigned to specific cellulases in the absence of protein identification data.

For normalization to intracellular protein, all mycelia from each 50 mL Avicel culture were harvested on day 7, filtered, washed with double-distilled water, and ground in liquid nitrogen. Protein was extracted with 1 mL of protein extraction buffer. Intracellular and extracellular protein concentrations were paired by culture flask and sampling time. The ratio was calculated using the nominal culture and extraction volumes as (Cout × 50 mL)/(Cin × 1 mL) and expressed as mg of extracellular protein per mg of intracellular protein. Three paired culture samples were included for each of WT, *Δkin-1*, *Ckin-1*, and *Δkin-1::kinesin*. This analysis was restricted to day 7 under Avicel conditions and was not applied to *Mclr-2* strains or sucrose cultures. Because residual insoluble Avicel was present in the samples, intracellular protein was used as a proxy for fungal biomass rather than a direct measure of mycelial dry weight or viability.

### 2.4. RNA Extraction and RT–qPCR

Samples for RT–qPCR were collected after 48 h of cultivation in Vogel’s medium containing 2% Avicel or 2% sucrose. Mycelia from 2 mL of culture were frozen in liquid nitrogen, mixed with 1 mL of TRIzol, and disrupted by bead milling. RNA was recovered by chloroform extraction, isopropanol precipitation, and washing with 75% ethanol, then dissolved in RNase-free water. Relative *cbh-1* and *clr-2* transcript levels were measured on an eQ9600 real-time PCR system (Eastwin Scientific Equipments Inc., Suzhou, China) using *β-actin* as the reference gene and calculated by the 2^−ΔΔCt^ method [33]. WT, *Δkin-1*, *WT::Mclr-2*, and *Δkin-1::Mclr-2* were compared under both carbon-source conditions, with three independent flask cultures per strain. Each target gene was normalized to WT within the corresponding carbon-source experiment. The original two-strain transcript comparison under Avicel conditions is reported in Section 3.2. The *clr-2* primers amplify a target sequence shared by the endogenous gene and the expression cassette and therefore do not distinguish endogenous from cassette-derived transcripts.

### 2.5. Cellulase Activity Assays

Culture supernatants collected at 168 h were used as crude enzyme preparations. Reactions were performed at 50 °C in 50 mM citrate–sodium citrate buffer (pH 4.8). Reducing sugars were measured by the dinitrosalicylic acid (DNS) assay using glucose as the standard [34]. The filter paper and carboxymethyl cellulose assays measure different aspects of cellulolytic activity; their results and unit conversions must be interpreted according to the definition of each assay [35].

For the filter paper activity (FPA) assay, a 6.0 × 0.8 cm strip of Whatman No. 1 filter paper was equilibrated in 1.5 mL of buffer, followed by addition of 0.5 mL of diluted enzyme and incubation for 60 min. For the CMCase assay, 1.5 mL of 1% sodium carboxymethyl cellulose was mixed with 0.5 mL of diluted enzyme and incubated for 30 min. Reactions were stopped by adding 2 mL of DNS reagent, heated in a boiling-water bath for 5 min, cooled, and diluted with 9 mL of water before measuring absorbance at 540 nm. For blanks, enzyme was added after the DNS reagent. One international unit (IU) of FPA or CMCase was defined as the amount of enzyme releasing reducing sugars equivalent to 1 μmol of glucose per minute under the specified assay conditions. Activities were expressed as IU/mL of culture supernatant.

For the pNPC hydrolysis assay, 50 μL of pNPC solution (1 mg mL^−1^) was mixed with 100 μL of diluted enzyme and incubated for 30 min. The pNPG hydrolysis assay followed the same procedure using 50 μL of pNPG solution (1.5 mg mL^−1^) as the substrate. Reactions were stopped with 150 μL of 1 M Na_2_CO_3_, and the released p-nitrophenol was measured at 420 nm. For blanks, the Na_2_CO_3_ was added before the enzyme. One IU was defined as the amount of enzyme releasing 1 μmol of p-nitrophenol per minute under the specified conditions. Activities were expressed as IU/mL of culture supernatant, not as specific activities normalized to protein concentration.

### 2.6. Saccharification of Pretreated Straw

*Miscanthus* straw, corn stover, and rice straw were pretreated with 0.25 M NaOH at 50 °C for 20 h. Each saccharification reaction contained 0.5 g of pretreated dry substrate, crude culture supernatant supplying 0.1 mg of total protein, and 0.1 mL of 2% ProClin 300. The volume was adjusted to 10 mL with 50 mM citrate–sodium citrate buffer (pH 4.8), and reactions were incubated at 50 °C and 150 rpm for 72 h. Samples were collected at 0, 0.5, 1, 2, 4, 8, 12, 24, 36, 48, 60, and 72 h, heated for 5 min, and centrifuged at 8000 rpm for 5 min. Reducing sugars were measured by the DNS assay. Yields were expressed as mg of reducing-sugar equivalents released per g of dry matter. Total protein loading was held constant across strains, but the preparations were not normalized to equal cellulase activity.

### 2.7. Replication and Statistical Analysis

Data are presented as means ± standard deviations (SD), with the unit of replication specified in the figure legends. Independent cultures of a selected strain are distinguished from independently obtained transformants. Statistical analysis used one-way ANOVA followed by Dunnett’s multiple-comparisons test against WT within the same assay, culture condition, and sampling time. RT–qPCR comparisons were performed separately for each target gene. All significance symbols indicate comparisons with WT: * *p* < 0.05, ** *p* < 0.01, *** *p* < 0.001, and **** *p* < 0.0001; ns, not significant. Tables S3 and S4 provide descriptive summaries only, without additional tests between non-WT strains.

All significance annotations in the figures use WT as the reference and do not test differences between the two *Mclr-2* strains or between complemented strains and *Δkin-1*. Unless a direct test is reported, comparisons between non-WT strains in the text therefore describe only the observed means or patterns. A nonsignificant difference from WT is not evidence of statistical equivalence. Branch measurements were obtained from selected axes within the displayed images, not from independent biological replicates, and inference is therefore limited to those images.

## 3. Results

### 3.1. Deletion of kin-1 Alters Growth and Morphology Without Reducing Biomass Under Sucrose Conditions

**Figure 1:**
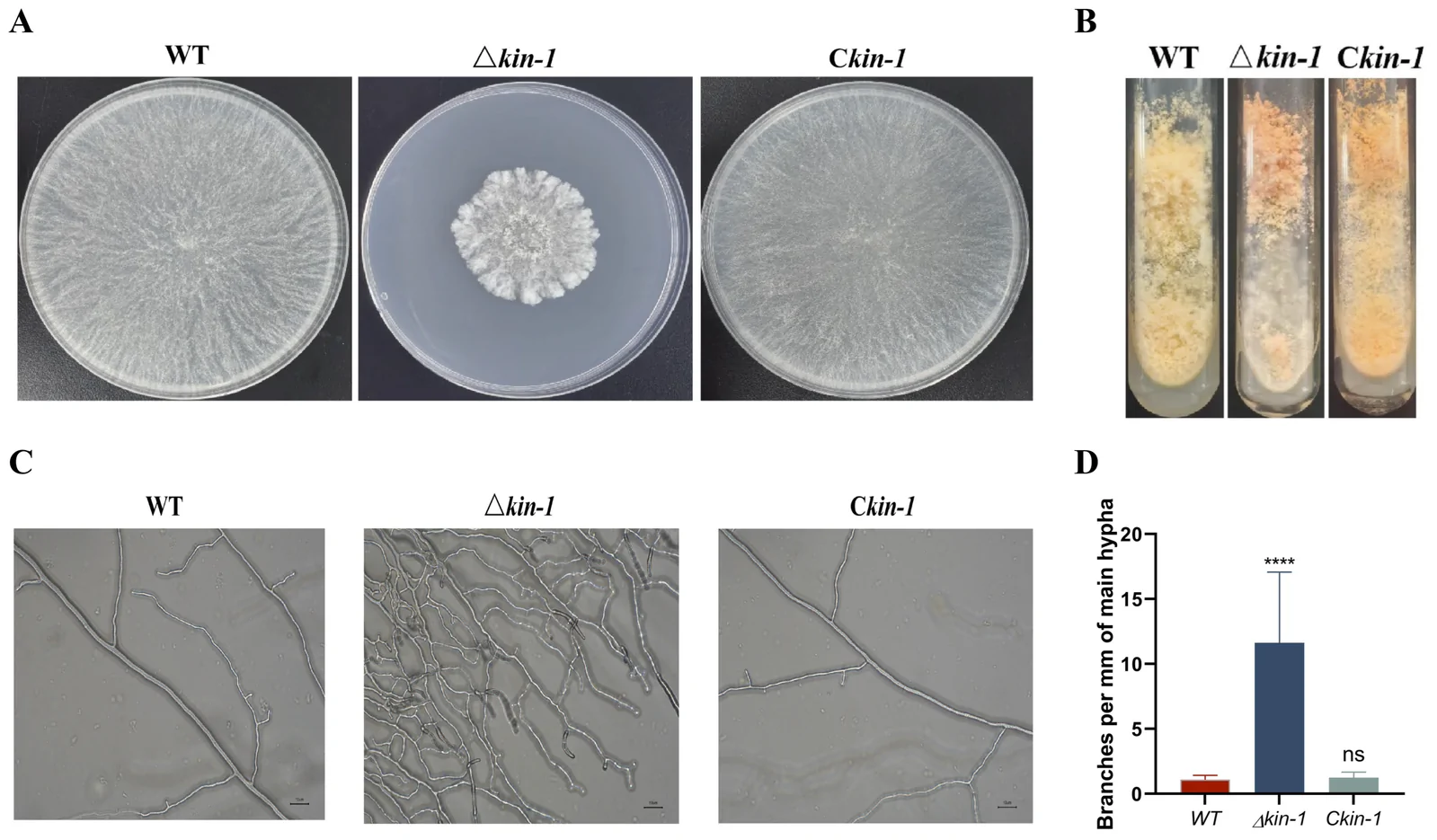
Colony and hyphal phenotypes of WT, *Δkin-1*, and *Ckin-1* on sucrose medium. (**A**) Colonies after 48 h at 28 °C on Vogel’s medium containing 2% sucrose. (**B**) Representative slant cultures after approximately 7 d. (**C**) Hyphal morphology after 12 h. (**D**) Added branch-density measurements, shown as supplied by the authors. Bars and error bars summarize observations within images as means ± SD; these observations are not independent biological replicates. *Ckin-1* is the strain complemented with the native gene. Statistical comparisons used one-way analysis of variance (ANOVA) followed by Dunnett’s multiple-comparisons test against WT within the same assay, culture condition, and sampling time. Symbols indicate comparisons with WT: **** *p* < 0.0001; ns, not significant.

On sucrose medium, *Δkin-1* formed compact colonies with less expansion than WT, whereas the colony appearance of *Ckin-1* more closely resembled WT (Figure 1A). Aerial hyphal growth in slant cultures differed among strains (Figure 1B). Hyphae of *Δkin-1* were more densely branched than the more extended hyphae of WT and *Ckin-1* (Figure 1C). The added branch-density plot describes selected main hyphal axes in the displayed images (Figure 1D); these observations cannot establish population-level differences among independent cultures. Hyphal morphology of independent clones and transformants is shown in Figure S8.

**Figure 2:**
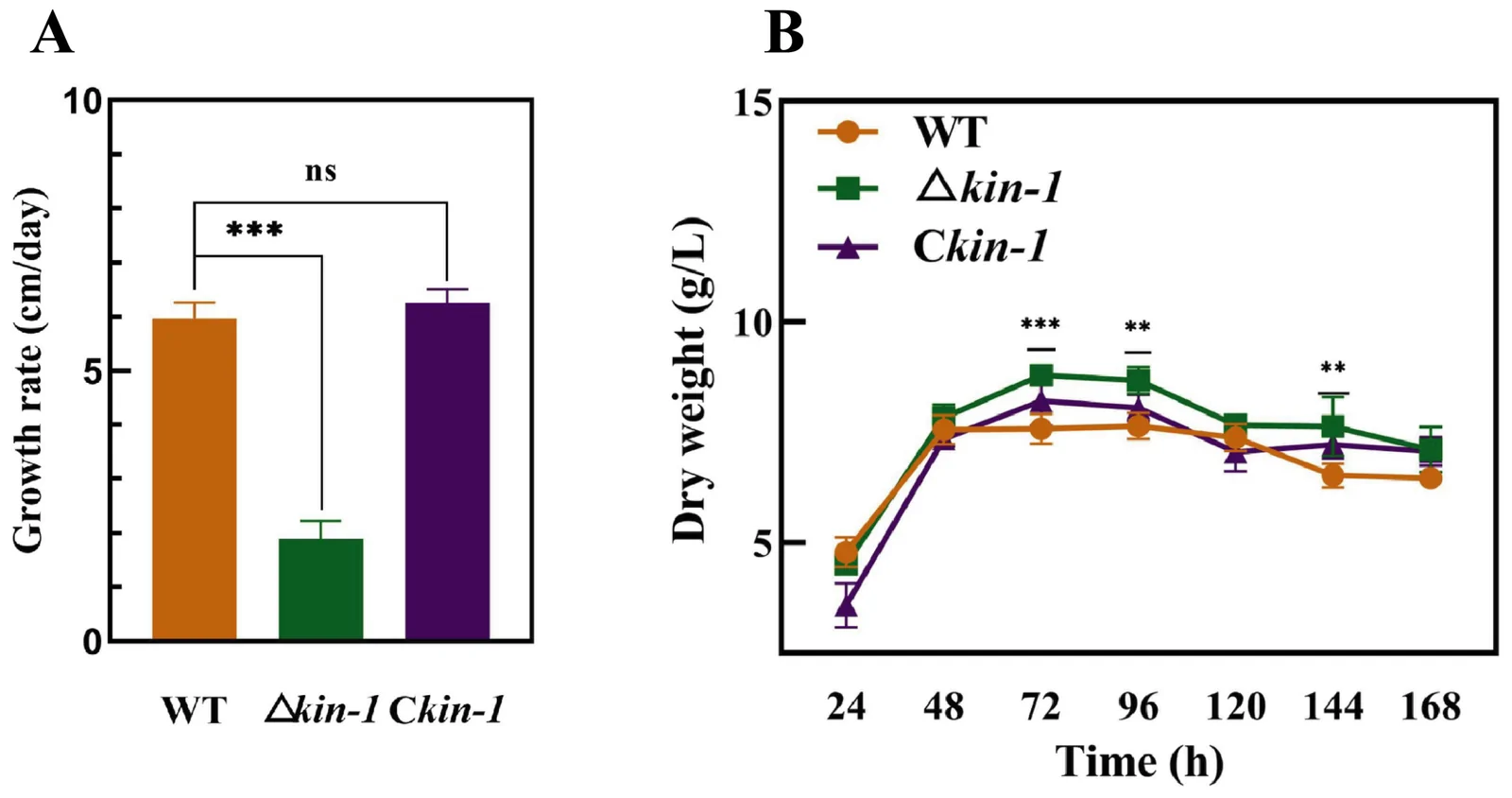
Growth rates and biomass of WT, *Δkin-1*, and *Ckin-1*. (**A**) Linear extension was measured every 24 h in race tubes containing Vogel’s medium with 2% sucrose at 28 °C, and growth rates are expressed as cm day^−1^. (**B**) Dry biomass was measured at 24–168 h in 24-well plates containing 3 mL of sucrose medium per well at 28 °C and 580 rpm. Data are means ± SD from three independent biological replicates. Comparisons used one-way ANOVA followed by Dunnett’s multiple-comparisons test against WT; biomass comparisons were performed separately at the indicated time points. ** *p* < 0.01; *** *p* < 0.001; ns, not significant.

Growth in race tubes was slower in *Δkin-1* than in WT, whereas *Ckin-1* did not differ significantly from WT in the reported comparison (Figure 2A). Biomass in sucrose liquid cultures showed a different pattern. The dry weight of *Δkin-1* was not generally reduced and was higher than that of WT at the annotated time points of 72, 96, and 144 h (Figure 2B). Thus, under these conditions, slower hyphal extension and compact colonies did not imply reduced biomass.

### 3.2. Deletion of kin-1 Reduces Extracellular Protein Accumulation and cbh-1/clr-2 Transcript Abundance Under Avicel Conditions

**Figure 3:**
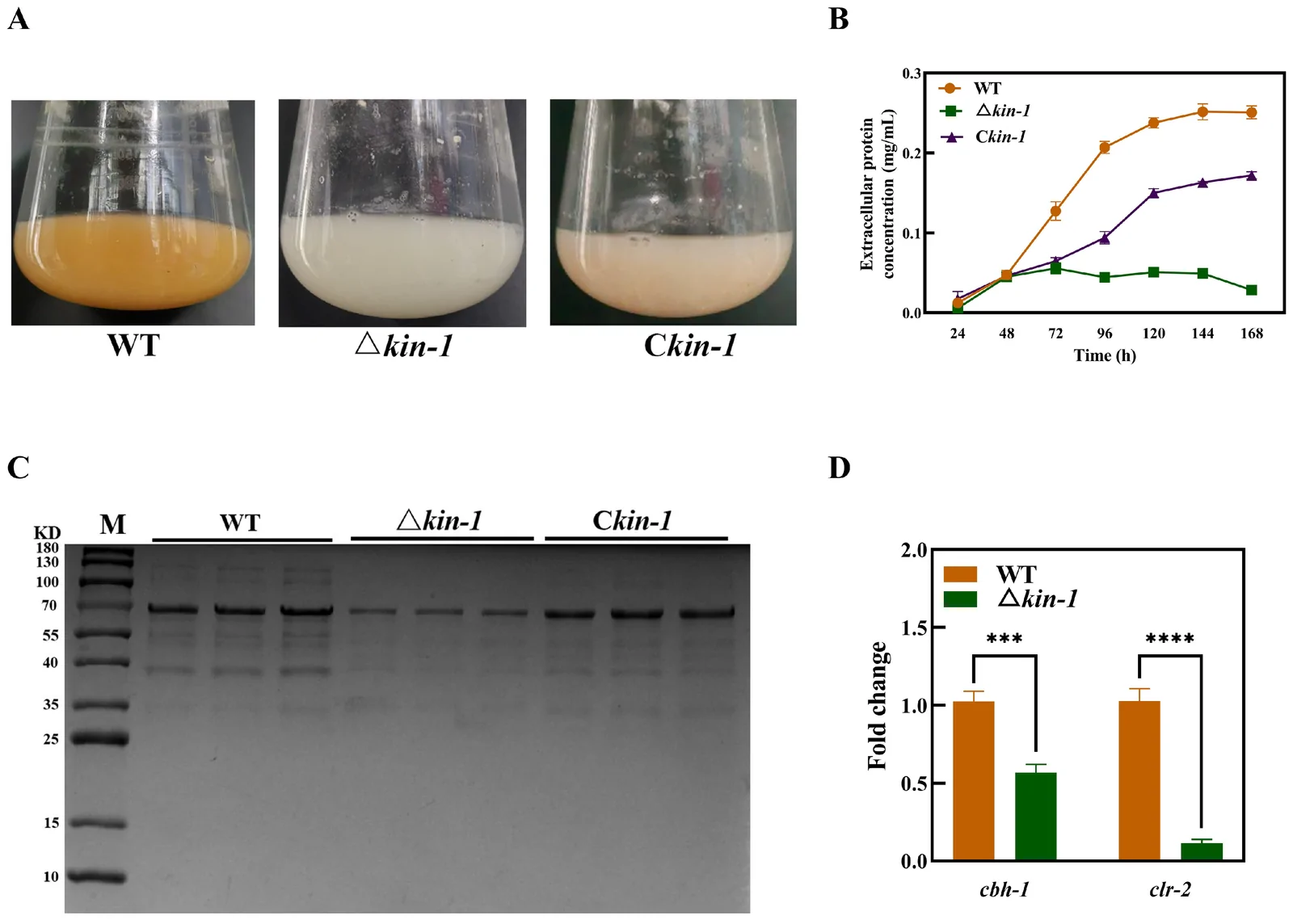
Culture appearance, extracellular protein accumulation, and cellulolytic transcripts in *kin-1* mutant strains. WT, *Δkin-1*, and *Ckin-1* were cultivated in 250 mL flasks containing 50 mL of Vogel’s medium with 2% Avicel at 28 °C and 220 rpm, with an initial inoculum of 10^6^ conidia mL^−1^. (**A**) Flask cultures after 168 h. (**B**) Extracellular protein concentrations measured every 24 h up to 168 h. (**C**) SDS–PAGE of prepared culture supernatant samples collected at 168 h; M, protein molecular-weight marker (kDa). (**D**) Original two-strain comparison of relative *cbh-1* and *clr-2* transcript abundance after 48 h under Avicel conditions, calculated by the 2^−ΔΔCt^ method using *β-actin* as the reference gene. The original plot, error bars, and annotations are retained; the added four-strain transcript dataset is shown separately in Figure 4E. Statistical comparisons used one-way ANOVA followed by Dunnett’s multiple-comparisons test against WT within the same assay, culture condition, and sampling time. RT–qPCR comparisons were performed separately for each target gene. Symbols indicate comparisons with WT: *** *p* < 0.001 and **** *p* < 0.0001.

The Avicel cultures differed markedly in appearance (Figure 3A). WT cultures were orange, *Δkin-1* cultures were predominantly white, and *Ckin-1* showed an intermediate appearance. These photographs provide only a qualitative comparison of cultural appearance; residual Avicel and conidial abundance were not quantified. Under Avicel conditions, extracellular protein accumulated progressively in WT but remained substantially lower in *Δkin-1* after the early sampling points (Figure 3B). *Ckin-1* showed recovery relative to the deletion strain, although its late-stage protein concentrations remained below WT. SDS–PAGE with equal volumes of culture supernatant showed weaker overall protein bands in *Δkin-1* and recovery of several major bands in *Ckin-1* (Figure 3C). Because the proteins in these bands were not identified, these changes are interpreted as alterations in the extracellular protein profile rather than direct measurements of individual cellulase abundance.

To determine whether this phenotype involved transcriptional changes, we measured *cbh-1* and *clr-2* transcript abundance after 48 h under Avicel conditions. Both were lower in *Δkin-1* than in WT, with a more pronounced reduction in *clr-2* (Figure 3D). Deletion of *kin-1* was therefore associated with reduced expression of a major cellulase gene and a central regulator of cellulose degradation. This comparison does not establish direct regulation by KIN-1. The reduction in *clr-2* prompted us to test whether constitutive expression of this regulator could restore extracellular enzyme production in the deletion background.

**Figure 4:**
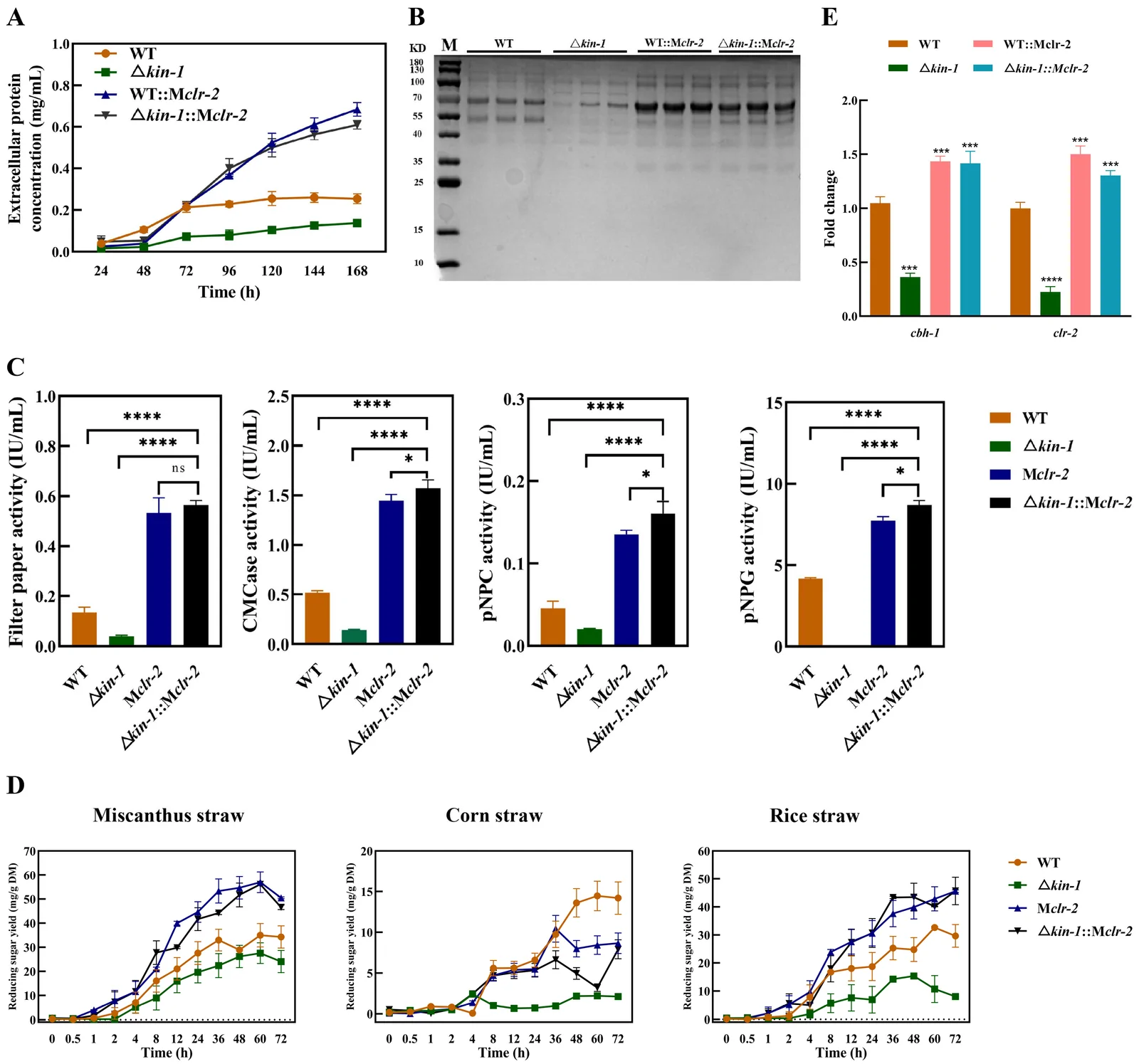
Effects of constitutive *clr-2* expression under Avicel conditions. WT, *Δkin-1*, *WT::Mclr-2*, and *Δkin-1::Mclr-2* were cultivated in Vogel’s medium containing 2% Avicel at 28 °C and 220 rpm. (**A**) Extracellular protein concentrations at 24–168 h. (**B**) SDS–PAGE of equal volumes of prepared culture supernatant samples collected at 168 h; M, protein molecular-weight marker (kDa). (**C**) FPA, CMCase, pNPC hydrolysis, and pNPG hydrolysis activities in culture supernatants collected at 168 h (IU/mL). (**D**) Reducing-sugar release during 72 h of hydrolysis of alkali-pretreated *Miscanthus* straw, corn stover, and rice straw at a total protein loading of 0.1 mg per reaction, expressed as mg g^−1^ dry matter. Enzyme activity and saccharification data are means ± SD (*n* = 3). *Mclr-2* in panels (**C**,**D**) denotes *WT::Mclr-2*. (**E**) Relative *cbh-1* and *clr-2* transcript abundance in the four strains, with each gene referenced to the corresponding WT control under this culture condition. These measurements do not distinguish endogenous *clr-2* transcripts from cassette-derived *clr-2* transcripts. Data in panel (**E**) are means ± SD from three independent cultures sampled at 48 h, normalized by the 2^−ΔΔCt^ method using *β-actin* as the reference gene. Statistical comparisons used one-way ANOVA followed by Dunnett’s multiple-comparisons test against WT within the same assay, culture condition, and sampling time. RT–qPCR comparisons were performed separately for each target gene. Symbols indicate comparisons with WT: * *p* < 0.05, *** *p* < 0.001, and **** *p* < 0.0001; ns, not significant.

### 3.3. Constitutive clr-2 Expression Increases Enzyme Production in Δkin-1 Under Avicel Conditions, with Substrate-Dependent Saccharification

Under Avicel conditions, the protein concentration curve of *Δkin-1::Mclr-2* exceeded those of *Δkin-1* and WT during late cultivation but remained below *WT::Mclr-2* at the final sampling points (Figure 4A). SDS–PAGE showed more prominent extracellular protein bands in both *Mclr-2* strains than in *Δkin-1* (Figure 4B). Thus, despite the absence of *kin-1*, the deletion strain retained the capacity to accumulate extracellular protein above WT levels after constitutive *clr-2* expression.

The added four-strain RT–qPCR comparison showed higher *clr-2* and *cbh-1* transcript values in both *Mclr-2* strains than in *Δkin-1* (Figure 4E). Mean *clr-2* values for WT, *Δkin-1*, *WT::Mclr-2*, and *Δkin-1::Mclr-2* were 1.00, 0.22, 1.50, and 1.30, respectively, and the corresponding *cbh-1* means were 1.05, 0.36, 1.43, and 1.42. These measurements support an association between the expression cassette and increased transcript abundance in the deletion background. However, the primers measure total target-gene transcripts and cannot isolate expression from the introduced cassette.

Mean cellulolytic enzyme activities were higher in *Δkin-1::Mclr-2* than in *Δkin-1* (Figure 4C). The statistical annotations in this panel indicate comparisons of each strain with WT and cannot establish significant differences between *Δkin-1::Mclr-2* and *Δkin-1* or between the two *Mclr-2* strains. The rankings of individual enzyme activities did not match that of total extracellular protein concentration. Constitutive *clr-2* expression was associated with recovery of several enzyme-production phenotypes in the deletion background, although the extent of recovery differed among assays.

At an equal total protein loading of 0.1 mg per reaction, reducing-sugar release depended on the straw substrate (Figure 4D). On *Miscanthus* and rice straw, enzyme preparations from both *Mclr-2* strains produced reducing-sugar release curves above those of WT and *Δkin-1*. On *Miscanthus*, *WT::Mclr-2* generally exceeded *Δkin-1::Mclr-2*, whereas the two cassette-bearing strains gave similar values at the final time point on rice straw. Corn stover showed a different ranking: WT had the highest final reducing-sugar release, and both *Mclr-2* strains were below WT. These descriptive comparisons concern hydrolysis at equal total protein loading and therefore cannot simply be explained by adding more protein from cultures with higher protein yields. Enzyme preparations from the *Mclr-2* strains did not improve hydrolysis on every substrate tested.

### 3.4. Constitutive clr-2 Expression Enhances Protein Production, Cellulase Activity, and Straw Saccharification Under Sucrose Conditions

The recovery of some endpoints under Avicel conditions raised a further question: can constitutive *clr-2* expression also sustain cellulolytic enzyme output under growth conditions without cellulose induction? To address this question, we replaced Avicel with sucrose as the culture carbon source and measured extracellular protein accumulation, cellulase activity, and the saccharification performance of the resulting culture supernatants.

**Figure 5:**
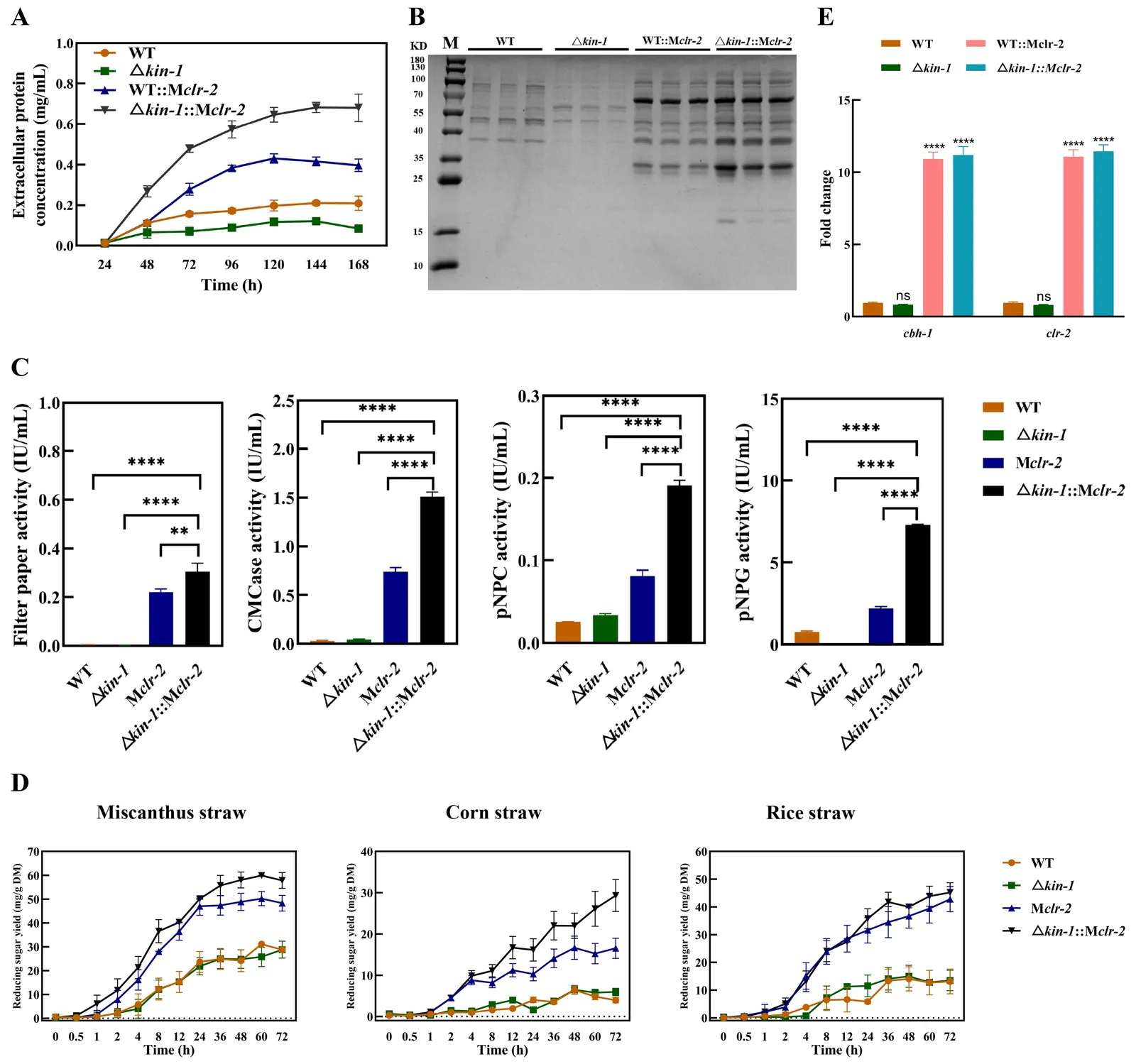
Effects of constitutive *clr-2* expression under sucrose conditions. WT, *Δkin-1*, *WT::Mclr-2*, and *Δkin-1::Mclr-2* were cultured in Vogel’s medium containing 2% sucrose at 28 °C and 220 rpm. (**A**) Extracellular protein concentrations from 24–168 h. (**B**) SDS–PAGE of prepared 168 h culture supernatant samples loaded at equal volumes; M, protein molecular-weight marker (kDa). (**C**) FPA, CMCase, pNPC hydrolysis activity, and pNPG hydrolysis activity in 168 h culture supernatants (IU/mL). (**D**) Reducing-sugar release during hydrolysis of alkali-pretreated *Miscanthus*, corn stover, and rice straw, with 0.1 mg total protein added per reaction and sampling up to 72 h, expressed as mg g^−1^ dry matter. Enzyme activity and saccharification data are means ± SD (*n* = 3). *Mclr-2* in C/D denotes *WT::Mclr-2*. (**E**) Relative transcript abundance of *cbh-1* and *clr-2*, normalized separately for each gene to the corresponding WT under sucrose conditions. This assay measures total *clr-2* transcripts rather than cassette-specific expression. Data in E are means ± SD from 3 independent cultures sampled after 48 h, normalized by the 2^−ΔΔCt^ method using *β-actin* as the reference gene. Statistical comparisons used one-way ANOVA followed by Dunnett’s multiple-comparisons test against WT within the same assay, culture condition, and sampling time. For RT–qPCR, comparisons were performed separately for each target gene. Symbols indicate comparisons with WT: ** *p* < 0.01 and **** *p* < 0.0001; ns, not significant.

When sucrose rather than Avicel was used as the culture substrate, *Δkin-1::Mclr-2* accumulated the highest extracellular protein concentration late in cultivation, followed by *WT::Mclr-2*, WT, and *Δkin-1* (Figure 5A). Thus, under this condition, the cassette-bearing strain in the deletion background exceeded its counterpart in the WT background. Both *Mclr-2* strains showed prominent extracellular protein bands, although band intensities and protein profiles differed (Figure 5B). Extracellular protein remained measurable in WT and *Δkin-1*; growth on sucrose did not eliminate protein release.

Transcript comparisons under sucrose conditions showed that *cbh-1* and *clr-2* values in both cassette-bearing strains were approximately 11 relative units, compared with approximately 0.8–1.0 in WT and *Δkin-1* (Figure 5E). This confirms increased target-gene transcript abundance in the engineered strains under culture conditions without Avicel. Because the experiments with each carbon source were normalized to their respective WT controls, these fold-change values cannot establish differences in absolute transcript levels between Avicel and sucrose conditions.

At 168 h of cultivation, mean FPA, CMCase, pNPC hydrolysis activity, and pNPG hydrolysis activity in *Δkin-1::Mclr-2* were all higher than those in *Δkin-1* and *WT::Mclr-2* (Figure 5C). The significance annotations in the figure indicate comparisons of each strain with WT; comparisons between non-WT strains are descriptive. The parental strains without the cassette had low enzyme activities, although residual activity remained detectable in some assays. These results indicate that constitutive *clr-2* expression enables relatively high cellulolytic enzyme production under sucrose conditions and that the observed effect depends on the *kin-1* background. This experiment did not test whether all components of carbon catabolite repression had been relieved.

At equal total protein input, the enzyme preparation from sucrose-grown *Δkin-1::Mclr-2* produced the highest mean reducing-sugar release curves on the 3 straw substrates tested, followed by *WT::Mclr-2* (Figure 5D). The curves for WT and *Δkin-1* were lower and broadly similar on *Miscanthus* and rice straw, whereas their values differed on corn stover. The size of the difference between the two cassette-bearing strains varied with the substrate. These data show differences in the hydrolytic performance of enzyme preparations from sucrose-grown cultures at matched protein input; they do not establish that the specific activity of every enzyme in the preparations was increased.

### 3.5. The T. reesei kin-1 Gene Alleviates Growth and Branching Defects in N. crassa Δkin-1

The *clr-2* experiments tested whether constitutive expression of this regulator could bypass some effects of *kin-1* deletion. To examine whether some KIN-1 functions are conserved between fungi, we complemented the *N. crassa Δkin-1* strain with the *kin-1* gene from the cellulase-producing fungus *T. reesei* and evaluated growth, morphology, extracellular protein accumulation, and cellulase activity.

**Figure 6:**
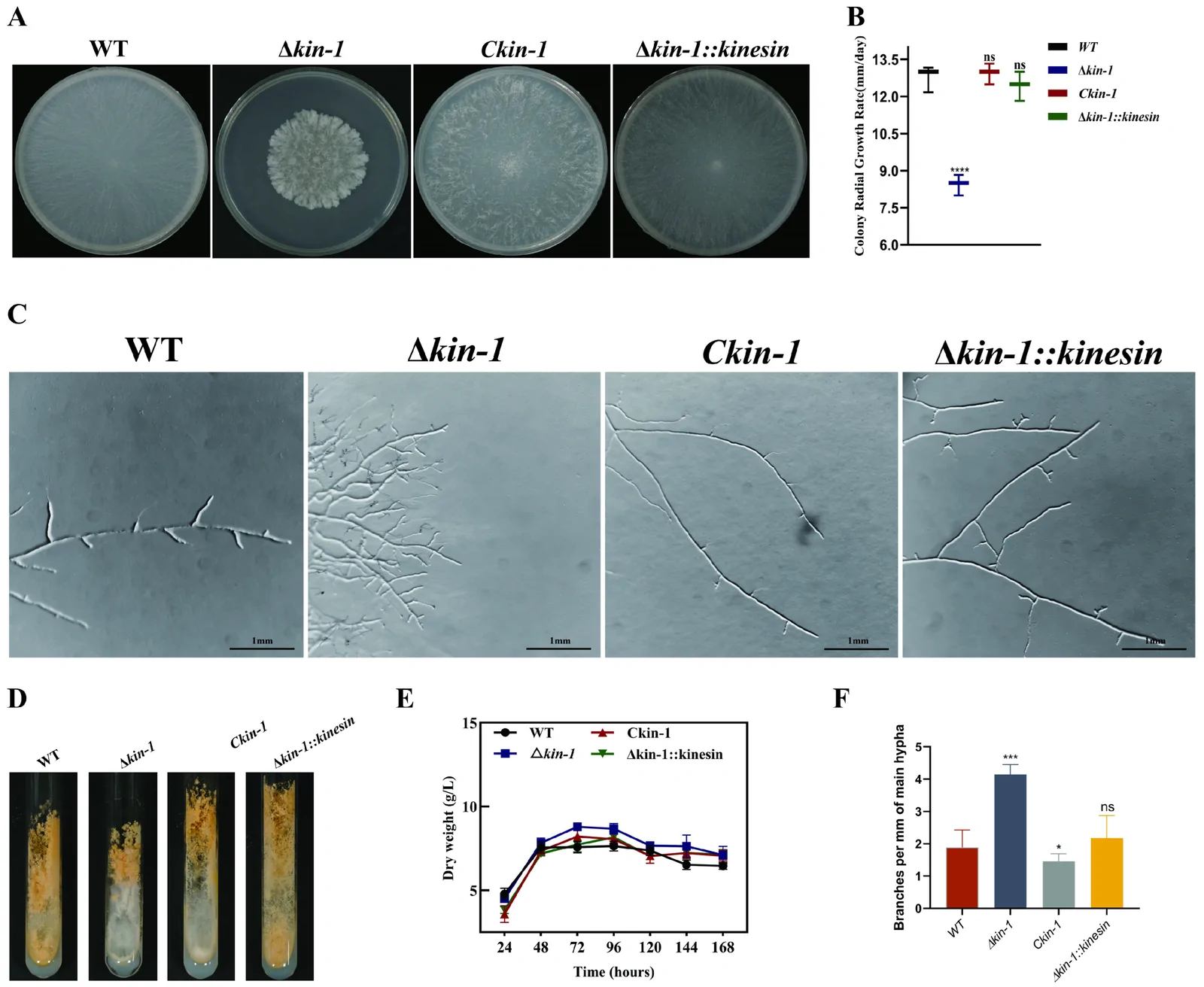
Growth-related phenotypes following heterologous kinesin complementation. WT, *Δkin-1*, *Ckin-1*, and *Δkin-1::kinesin* were compared under the sucrose growth conditions described in Section 2.2. (**A**) Colonies after 48 h of cultivation. (**B**) Radial growth rates. (**C**) Hyphal morphology after 12 h of cultivation. (**D**) Slant cultures after approximately 7 d. (**E**) Dry biomass from 24–168 h. (**F**) Additional branch density measurements, presented in the author-supplied panel as means ± SD of observations within images. These observations are not independent biological replicates. *Ckin-1* is the native complementation strain; *Δkin-1::kinesin* carries the *T. reesei* gene. Statistical comparisons used one-way ANOVA followed by Dunnett’s multiple-comparisons test against WT within the same assay, culture condition, and sampling time. Symbols indicate comparisons with WT: * *p* < 0.05, *** *p* < 0.001, and **** *p* < 0.0001; ns, not significant.

We separately compared WT, *Δkin-1*, *Ckin-1*, and *Δkin-1::kinesin* to test functional substitution. The *T. reesei* gene alleviated the compact-colony phenotype of *Δkin-1*, producing colonies more similar in appearance to WT and *Ckin-1* (Figure 6A). The radial colony growth rate increased toward the WT value (Figure 6B). Neither complemented strain differed significantly from WT in the reported growth-rate comparisons, although these comparisons were not formal equivalence tests.

Microscopy showed sparser branching and more extended hyphae in the heterologously complemented strain than in *Δkin-1* (Figure 6C). The aerial hyphal growth pattern in slant cultures was also partly restored (Figure 6D). Under sucrose conditions, the dry-biomass curves of the 4 strains largely overlapped from 24–168 h (Figure 6E). The additional branch density panel describes selected main hyphal axes within these images (Figure 6F), rather than independent cultures. Supplementary colony and hyphal microscopy images of independent isolates (Figures S5 and S8) provide qualitative comparisons of the morphological phenotypes.

### 3.6. Extracellular Protein Accumulation and Cellulase Activity Following Heterologous Complementation

**Figure 7:**
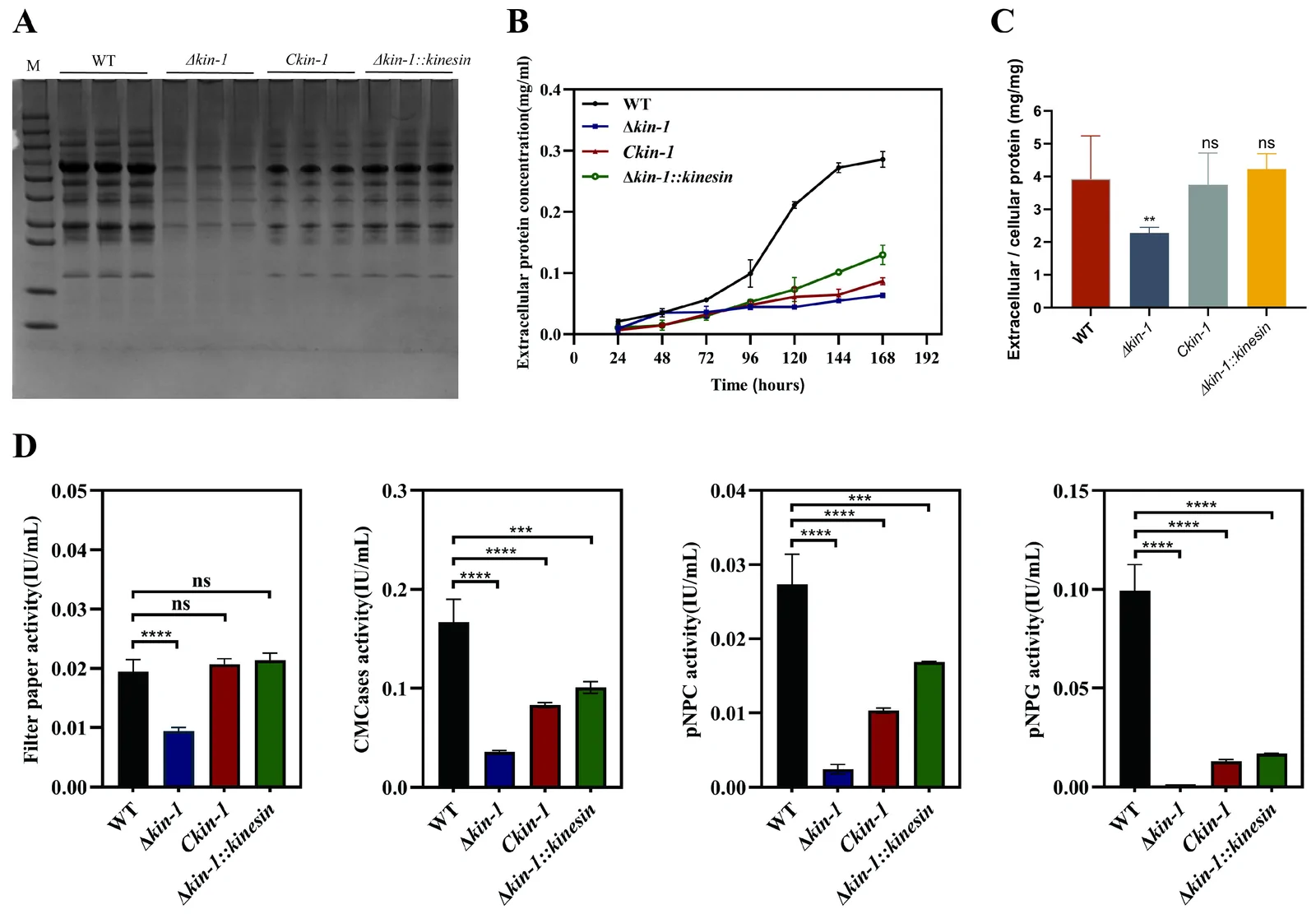
Extracellular protein and cellulase assays following heterologous complementation under Avicel conditions. WT, *Δkin-1*, *Ckin-1*, and *Δkin-1::kinesin* were cultured in Vogel–2% Avicel medium at 28 °C and 220 rpm. (**A**) SDS–PAGE with the molecular-weight marker and samples from the same gel; the uncropped gel image and lane map are provided in Supplementary Data S2. (**B**) Extracellular protein-concentration time courses from 24–168 h. (**C**) The extracellular/intracellular protein mass ratio on day 7, calculated from paired measurements from the same culture as (extracellular protein concentration × 50 mL)/(intracellular protein concentration × 1 mL). Intracellular protein serves as a proxy for biomass in this experiment. Data are means ± SD from 3 paired cultures per strain; a descriptive summary is provided in Table S3. (**D**) FPA, CMCase, pNPC, and pNPG activities on day 7 (IU/mL), with the existing panel retained. Statistical comparisons used one-way ANOVA followed by Dunnett’s multiple-comparisons test against WT within the same assay, culture condition, and sampling time. Symbols indicate comparisons with WT: ** *p* < 0.01, *** *p* < 0.001, and **** *p* < 0.0001; ns, not significant.

We next examined protein- and cellulase-related phenotypes in the complemented strains under Avicel conditions. The SDS–PAGE panel includes the molecular-weight marker and sample lanes from the same gel and shows more prominent extracellular protein bands in *Ckin-1* and *Δkin-1::kinesin* than in *Δkin-1* (Figure 7A; the uncropped image is provided in Supplementary Data S2). The protein-concentration time courses showed greater extracellular protein accumulation in both complemented strains than in *Δkin-1*, although their late-culture values remained below WT (Figure 7B). This comparison reflects protein amounts per unit volume of culture supernatant.

We then normalized the extracellular protein mass on day 7 to the intracellular protein amount from the same culture (Figure 7C). The mean ratios ± SD for WT, *Δkin-1*, *Ckin-1*, and *Δkin-1::kinesin* were 3.92 ± 1.32, 2.27 ± 0.17, 3.75 ± 0.96, and 4.23 ± 0.46 mg/mg, respectively (3 paired cultures per strain; Table S3). The normalized extracellular protein levels of both complemented strains were restored to near-WT levels, with neither differing significantly from WT in the reported Dunnett comparisons. This endpoint reflects extracellular protein accumulation relative to intracellular protein and cannot distinguish active secretion from protein release caused by cell damage.

The updated enzyme activity records from day 7 of Avicel cultivation indicate that each assay endpoint requires separate evaluation (Table S4). For pNPC hydrolysis activity, the recorded means ± SD for WT, *Δkin-1*, *Ckin-1*, and *Δkin-1::kinesin* were 0.02743 ± 0.00405, 0.00242 ± 0.00063, 0.10315 ± 0.00361, and 0.01666 ± 0.00037 IU/mL, respectively, with three numerical records per group. The recorded mean for *Ckin-1* was higher than that for WT, whereas that for *Δkin-1::kinesin* was lower than WT but higher than *Δkin-1*. For pNPG hydrolysis activity, the recorded means for *Ckin-1* and *Δkin-1::kinesin* were 0.01293 and 0.01687 IU/mL, respectively, both lower than the WT value of 0.09908 IU/mL and higher than the *Δkin-1* value of 0.00162 IU/mL. Comparisons across phenotypes and the complete numerical summary are provided in Tables S2 and S4, respectively.

## 4. Discussion

### 4.1. KIN-1 Links Growth Morphology to Extracellular Enzyme Production, but Hyphal Extension and Biomass Are Distinct Measures

Deletion of *kin-1* altered growth morphology without a corresponding reduction in dry biomass under sucrose conditions. Compact colonies, slower hyphal extension, and dense branching coexisted with maintained or increased biomass at some time points. These observations are consistent with the roles of conventional kinesin in polarized growth and hyphal tip organization [22,23]. Actin- and microtubule-dependent processes jointly maintain the tip growth apparatus [36]. When this organization is altered, the observation of more branches does not necessarily imply a greater number of efficient secretion sites.

The relationship between morphology and enzyme production is not uniform across mutants or carbon sources. In *T. reesei*, the effects of *rac1* deletion on morphology and cellulase activity depend on the conditions [37], while disruption of *gul1* also alters morphology and production traits [38]. Together with the differing phenotypes of *N. crassa gul-1* and *A. niger racA* [29,30], these studies do not support a general rule that increased branching necessarily enhances secretion. Because the culture conditions differ, the dry-biomass experiment in sucrose cultures cannot be used to normalize the protein-concentration time courses under Avicel conditions. The additional paired measurements on day 7 in Avicel cultures address this mismatch for the complemented strains by using intracellular protein as the normalization denominator. The mean values suggest that differences in cellular material may partly account for the lower extracellular protein concentrations per unit volume in the complemented cultures. This endpoint ratio cannot distinguish secretion from protein synthesis, extracellular stability, or release caused by cell damage.

### 4.2. Constitutive clr-2 Expression Can Provide a Functional Bypass but Does Not Establish Direct Transcriptional Regulation by KIN-1

The reductions in *clr-2* and *cbh-1* transcripts suggest that altered gene expression contributes to the extracellular protein phenotype. Recovery following introduction of the constitutive expression cassette indicates that *Δkin-1* retains the capacity to produce cellulolytic enzymes when the corresponding regulatory program is activated. Constitutive *clr-2* expression can also bypass the enzyme-production defect in a VIB1-deletion background, providing a precedent for this type of genetic response [12]. Analyses of CLR-1/CLR-2 target genes and studies of constitutive CLR-2 activity suggest that multiple extracellular enzyme genes, rather than a single protein, may be affected [10,11].

The additional transcript measurements across four strains show higher total *clr-2* and *cbh-1* transcript abundance in the cassette-bearing strains, particularly relative to *Δkin-1* under Avicel conditions and to the parental strains without the cassette under sucrose conditions. These assays do not distinguish transcription from the endogenous *clr-2* locus from that arising from the introduced cassette, nor did they measure CLR-2 protein abundance, promoter occupancy, or localization. Direct interactions between KIN-1 and transcriptional regulators were not tested in this study. Transport defects could indirectly affect transcription by altering nutrient sensing, cellular organization, or stress physiology. Cellodextrin transporters and CLP1 participate in induction, so transport-related and transcriptional phenotypes are not necessarily mutually exclusive [5,7]. Proteomic and phosphoproteomic studies have also identified regulatory changes beyond alterations in transcript abundance [39]. These findings support a genetic bypass of part of the enzyme-production phenotype but do not place KIN-1 exclusively within a single linear KIN-1–CLR-2 pathway.

### 4.3. Carbon Source and Enzyme-Mixture Composition Jointly Influence Saccharification Phenotypes

The observed ranking of *WT::Mclr-2* and *Δkin-1::Mclr-2* changed with the carbon source. Under Avicel conditions, the cassette-bearing strain in the WT background accumulated more total extracellular protein, whereas enzyme activity measurements showed assay-dependent patterns. Under sucrose conditions, *Δkin-1::Mclr-2* had the highest mean protein concentration and enzyme activity values. These descriptions do not imply that differences between the two cassette-bearing strains were statistically tested, because the tests annotated in the figures all used WT as the control. Total extracellular protein alone therefore did not predict the observed cellulolytic enzyme output. Carbon-source-dependent transcriptional programs [14,40] and differences in enzyme-mixture composition [41] are possible explanations, but the proteins were not identified and their relative proportions were not quantified in this study.

The results under sucrose conditions are consistent with studies using regulatory engineering to support cellulase production without adding insoluble inducers. In *T. reesei*, XYR1 regulates hydrolytic enzyme expression [42], and the identification and functional characterization of ACE3 established its role in this regulatory program [43,44]. Engineering ACE3 or combining modified regulatory components can enable cellulase production under noninducing conditions [45,46,47]. These studies provide a basis for comparison, but they do not demonstrate that *Δkin-1::Mclr-2* has the same molecular regulatory network. Inducer-independent production does not necessarily mean that carbon catabolite repression is completely absent. Studies of STP1/CRT1 and CRT1 variants also link substrate uptake with induction in *T. reesei*, with effects depending on the transporter and experimental conditions [48,49].

The corn-stover results under Avicel conditions limit any broad conclusion that lignocellulose hydrolysis was improved across all substrates. At the same total protein input of 0.1 mg, the final reducing-sugar yield from corn stover remained higher with WT than with enzyme preparations from either *Mclr-2* strain. Higher FPA, CMCase, or p-nitrophenyl-substrate hydrolyzing activity therefore does not guarantee better performance on this pretreated straw substrate. Quantitative secretome studies in *N. crassa* have shown that reconstituted mixtures of the major cellulases do not fully reproduce the activity of crude secreted-protein mixtures on microcrystalline cellulose [41], while studies using crop residues show that the enzyme repertoire produced varies with the substrate [40]. Enzyme proportions, accessory proteins, and substrate accessibility may all contribute, but these factors were not measured here. Combining equal-total-protein and volume-based comparisons in regulatory-engineering studies [46] controls the protein input but does not ensure equal amounts of active cellulases or establish the mechanisms underlying different hydrolysis curves.

### 4.4. Heterologous Complementation Supports Conserved Motor Function, but Recovery Depends on the Phenotype

In the *N. crassa* deletion background, the *T. reesei* gene alleviated colony and branching abnormalities and restored the radial growth rate toward the WT value. The donor gene therefore substituted for several functions of the recipient KIN-1. This functional result complements comparative studies of kinesin repertoires and motor-dependent transport [24,50], but the demonstrated conservation is limited to the donor and recipient tested here. It neither establishes that kinesins are interchangeable across all filamentous fungi nor shows that they have identical cargo specificities.

Motor function depends on a coordinated transport system. In *A. nidulans*, conventional kinesin contributes to dynein accumulation at microtubule plus ends [51]; in *N. crassa*, perturbing dynein affects hyphal polarity and Spitzenkörper organization [52]. Delivery of Rab11 vesicles involves cooperation among kinesin-1, myosin-5, and dynein [25], and further studies of the HUM complex and myosin V have clarified mechanisms of coordination and compensation among motors [53,54]. These studies suggest that reintroducing kinesin may restore some functions within a broader transport network. Direct cargo tracking or live-cell localization will be needed to determine which specific processes are restored in the strains studied here.

Extracellular protein accumulation per unit volume was not restored to WT levels, indicating that the extent of recovery differs among endpoints. The extracellular protein concentration of the native complementation strain *Ckin-1* also remained below WT, so the residual differences cannot be attributed solely to differences in the *T. reesei* protein. In the updated numerical records for pNPC activity, the mean for *Ckin-1* was higher than WT, whereas that of the heterologously complemented strain remained lower than WT. The recorded mean pNPG activities of both complemented strains were lower than WT, while the blank-subtraction records for FPA and CMCase still require verification; recovery therefore cannot be classified uniformly across enzyme activities (Table S4). By contrast, on day 7 of cultivation under Avicel conditions, extracellular protein normalized to intracellular protein was restored to near-WT levels in both complemented strains. Expression level, insertion-site context, and differences in enzyme composition may all affect individual endpoints. Secretion stress and constraints on protein folding and transport can also affect extracellular protein production [55]. Images of independent isolates provide additional qualitative support, but the main quantitative measurements were still performed on selected strains. Recovery should therefore be described separately for each phenotype, culture condition, and normalization basis (Table S2).

### 4.5. Scope of the Evidence and Further Experiments

The available evidence links *kin-1* deletion to altered growth morphology, reduced cellulolysis-related transcription, and lower extracellular enzyme production, and shows recovery of some phenotypes following regulatory bypass or heterologous complementation. Several limitations remain. Intracellular protein is used here as a proxy for cellular material; differences in extraction recovery or protein content per unit biomass could affect the ratio. Cell viability and cell lysis were not measured. Thus, neither the volume-based measurements nor the normalized endpoint can distinguish active secretion, synthesis, degradation, and release caused by cell damage. The sequencing records cover only parts of the introduced constructs, including the P*ccg-1*–*clr-2* junction and part of the *T. reesei* gene, and do not establish that the full-length construct is error-free in every experimental isolate.

Further studies should measure cell viability and lysis in paired cultures, verify the uncovered construct regions and candidate sequence differences, and quantify expression of the cassette and kinesin in independently reconstructed strains. Branching measurements should also encompass independent cultures rather than selected image fields alone. Secretome profiling and saccharification experiments performed at matched enzyme activity would help identify the enzyme components responsible for differences that persist at equal total protein input. Combining these approaches with live-cell tracking of secretory carriers and motor localization will be needed to distinguish direct transport functions from indirect regulatory consequences.

## 5. Conclusions

Deletion of *kin-1* disrupted colony expansion and hyphal organization in *N. crassa* without reducing dry biomass under sucrose culture conditions. Under Avicel conditions, the deletion was associated with lower extracellular protein per unit volume and lower *cbh-1*/*clr-2* transcript abundance. Constitutive *clr-2* expression increased the measured transcript levels and bypassed some enzyme-production defects; at equal total protein input, hydrolytic performance depended on the carbon source and substrate. The *T. reesei* gene alleviated growth-related defects. The updated enzyme activity records indicate that each catalytic activity should be evaluated separately and that recovery of one endpoint cannot be extrapolated to all cellulase activities. On day 7 of cultivation under Avicel conditions, extracellular protein normalized to intracellular protein was restored to near-WT levels in both complemented strains. These results support phenotype-dependent functional substitution; the connection between kinesin-dependent transport and cellulolytic regulation still requires direct mechanistic experiments.

## Figures and Tables

**Table 1:** Experimental strains and their uses.

Strain	Genetic Features	Use or Supporting Records
WT	*N. crassa* FGSC 2489 (OR74A background)	Parental control; *kin-1* locus NCU09730
*Δkin-1*	*kin-1* open reading frame replaced with *hph*	Deletion strain; Figure S1
*Ckin-1*	*Δkin-1* carrying the native *kin-1* genomic region	Native complementation; Supplementary Method S2 and Figure S4
*WT::Mclr-2*	WT carrying the P*ccg-1*–*clr-2* expression cassette at *his-3*	Parent carrying the P*ccg-1* expression cassette; Figure S2
*Δkin-1::Mclr-2*	*Δkin-1* carrying the P*ccg-1*–*clr-2* expression cassette at *his-3*	Progeny with constitutive expression; Figure S2
*Δkin-1::kinesin*	*Δkin-1* carrying the kinesin gene from *T. reesei* QM6a	TRIREDRAFT_55060/Gene ID 18485786; Figure S3

## Data Availability

Additional numerical data, sequencing records, and uncropped gel images are provided in Supplementary Data S1–S4. Other data supporting this study are available from the corresponding author upon reasonable request.

## Supplementary Materials

The following supporting information can be downloaded at: https://www.mdpi.com/article/10.3390/jof12100762/s1; Supplementary Methods S1–S7: strain construction and screening, media and culture formulations, protein normalization, image measurements, and sequence records and coverage; Figure S1: construction and PCR screening of Δ*kin-1*; Figure S2: construction and PCR screening of Δ*kin-1::Mclr-2*; Figure S3: construction scheme and PCR screening of Δ*kin-1::kinesin*; Figure S4: PCR screening for native *kin-1* complementation; Figure S5: colony morphology of independent isolates; Figure S6: extracellular protein banding patterns of independent isolates; Figure S7: partial sequencing-read coverage of construct reference sequences; Figure S8: hyphal morphology of independent clones and transformants; Table S1: oligonucleotide sequences; Table S2: phenotypic changes following native complementation, *clr-2* expression, and heterologous complementation; Table S3: descriptive summaries of protein normalization, qPCR, and *Mclr-2* enzyme activities; Table S4: updated enzyme activity records for the complementation series. Supplementary Data S1 contains reference sequences, sequencing reads, and coverage records; Supplementary Data S2 contains uncropped gel images and lane maps; Supplementary Data S3 contains raw numerical data and image-annotation records for the additional measurements; Supplementary Data S4 contains the updated enzyme activity source workbook.
