# Supplementary material for: KIN-1 Function in Neurospora crassa Revealed by Constitutive clr-2 Expression and Cross-Species Complementation
Source: J Fungi (Basel). 2026 Oct 10;12(10):762. doi: 10.3390/jof12100762 (PMC13705841; doi:10.3390/jof12100762)
Supplement: Supplementary file 1 [file jof-12-00762-s001.zip › KIN1_Supplementary_Materials_English.pdf]

Journal of *Fungi*

Supplementary Materials

Investigating the Function of KIN-1 in *Neurospora crassa* through Constitutive *clr-2* Expression and Cross-Species Complementation

These supplementary materials describe strain construction and screening, medium formulations, additional protein and morphological measurements, sequencing read coverage, and the associated figures and tables. Sequence records, uncropped gels, and numerical data are provided in Supplementary Data S1, S2, and S3, respectively. The donor for heterologous complementation was *Trichoderma reesei* QM6a, whose *kin-1* has Gene ID 18485786; the *kin-1* gene in *N. crassa* is NCU09730.

Supplementary Methods

S1. Construction and Screening of *Δkin-1*

The complete open reading frame of *N. crassa kin-1* was replaced with the hygromycin resistance marker *hph*. The upstream and downstream homology arms were amplified from WT genomic DNA using *kin-1* 5′ flank F/R and *kin-1* 3′ flank F/R, respectively. The *hph* fragment was amplified from pSK, and the vector backbone was amplified using BG *kin-1* F/R. The fragments were joined by Gibson-type assembly at 50 °C for 20 min, followed by propagation in *Escherichia coli* DH5α. Multifragment assembly used 20–40 ng of vector backbone, with the fragments added at equimolar ratios. Primer sequences are listed in Table S1.

Strains were grown on Vogel's–2% sucrose medium at 28 °C, first in the dark for 16–24 h and then in the light for 4–6 d to obtain conidia. Conidia were harvested, washed with ice-cold 1 M sorbitol, and adjusted to 10^9 conidia mL^{-1}. A 100 µL aliquot of the conidial suspension was mixed with 10 µL of linearized DNA; the recorded DNA concentration was at least 300 ng μL^{-1}. Electroporation was performed in a 1 mm cuvette at 1.5 kV, 25 µF, and 200 Ω. After adding 1 mL of sorbitol, the suspension was added to Vogel's–FIGS agar medium and incubated at 28 °C in the dark for 2–4 d. Hygromycin B was used at 200 µg mL^{-1} for selection.

Candidate colonies were transferred to sucrose slants and screened by PCR. Candidate strains were purified by sporulation on water agar, isolation of single colonies, and repeated screening. In Figure S1, candidates T1–T7 were scored as positive based on detection of the diagnostic *hph* amplicon and absence of the WT *kin-1* product. These were the recorded screening criteria; endpoint PCR images alone cannot establish copy number or exclude all ectopic integrations.

S2. Construction of the Native Complementation Strain *Ckin-1*

A WT genomic fragment containing the complete *kin-1* open reading frame and its flanking regulatory sequences was amplified using *kin-1* F/R and assembled with the linearized pLN-Ihis backbone. The construct was integrated at the *his-3* locus. The *his-3*::*kin-1* parent was crossed with a *Δkin-1* parent of the opposite mating type on crossing medium at room temperature for approximately 3 weeks. Mature ascospores were heat-shocked at 60 °C for 30 min and plated on Vogel's–2% sucrose medium containing 200 µg mL^{-1} hygromycin B. Individual germinated ascospores were transferred, and the endogenous *kin-1* and complementation loci were examined. The selected progeny were designated *Ckin-1*. PCR screening of the native complementation candidates is shown in Figure S4.

P1 (Vkin-1 5′ flank F and Vkin-1 R) was used to detect the intact endogenous *kin-1* locus, with an expected product of 2283 bp in WT. P2 (VpLN-Ihis F/R) was used to detect the complementation-associated region, with an expected product of 4543 bp after integration and a product of approximately 1900 bp in WT and *Δkin-1*. Candidates T1–T5 lacked the target P1 product but yielded the expected P2 product, consistent with the expected complemented genotype (Figure S4).

S3. Construction and Screening of *Δkin-1*::*Mclr-2*

The WT::*Mclr-2* parent carried a *clr-2* expression cassette driven by the constitutive *ccg-1* promoter and integrated at the *his-3* locus. This strain was crossed with a *Δkin-1* strain of the opposite mating type, and progeny carrying both the deletion background and the expression cassette were selected. Figure S2 shows the construction and crossing scheme and the diagnostic PCR results for candidates T1–T5. Vkin-1 F/R detected the 1041 bp WT product, whereas VMclr-2 F/R detected the recorded 1714 bp cassette-associated product. T1–T3 are marked as selected candidates in the supplied figure. *Mclr-2* is an abbreviated designation for the constitutive expression strain and does not imply an alteration in the amino acid sequence of CLR-2.

S4. Heterologous Complementation with *T. reesei kin-1*

The heterologous donor was *Trichoderma reesei* QM6a, whose *kin-1* corresponds to Gene ID 18485786. The corresponding *N. crassa* gene is annotated as NCU09730 in the OR74A reference genome. The donor gene was introduced into the *N. crassa Δkin-1* background to obtain *Δkin-1*::*kinesin*. The expression unit in Figure S3A is labeled with the *tef1* promoter, kinesin open reading frame, *cyc1* terminator, and flanking his locus sequences. Figure S3B compares the diagnostic PCR bands of WT and *Δkin-1*::*kinesin*.

In the schematic, the P1 products of WT and the complemented strain are labeled as 1900 bp and 4589 bp, respectively, and the P2 products as 5530 bp and 3934 bp, respectively. These sizes are taken from the supplied screening figure; primer identities and expected products still need to be checked against the actual construct sequence. Growth, biomass, protein, and enzyme activity phenotypes were measured as described in the main text. Growth results are presented in Figure 6, and extracellular protein and enzyme activity results in Figure 7.

S5. Media and Routine Culture Formulations

LB medium contained 10 g tryptone, 5 g yeast extract, and 10 g NaCl per liter; solid plates additionally contained 18 g agar per liter. The medium was autoclaved at 121 °C for 20 min. Water agar contained 2% agar. The 50× Vogel's stock solution contained, per liter, 130 g sodium citrate dihydrate, 126 g KNO_3, 144 g $(NH_4)H_2PO_4$, 10 g $MgSO_4{\cdot}7H_2O$, 80 g KH_2PO_4, 5 g $CaCl_2{\cdot}2H_2O$, 5 mL trace element stock solution, and 2.5 mL biotin stock solution.

The trace element stock solution contained, per 100 mL, 5 g citric acid monohydrate, 5 g $ZnSO_4{\cdot}7H_2O$, 1 g $Fe(NH_4)_2(SO_4)_2{\cdot}6H_2O$, 0.25 g $CuSO_4{\cdot}5H_2O$, 0.05 g $MnSO_4{\cdot}H_2O$, 0.05 g H_3BO_3, and 0.05 g $Na_2MoO_4{\cdot}2H_2O$. The biotin stock solution was prepared by dissolving 1 mg biotin in 10 mL water. Single-strength Vogel's medium was prepared with 8 mL of the 50× stock solution per 400 mL final volume; solid medium contained 2% agar. The carbon source was 2% (w/v) sucrose or Avicel, as described in the main text.

The FIGS stock solution contained 20 g L-sorbose, 0.5 g D-fructose, and 0.5 g D-glucose per 100 mL and was sterilized by filtration through a 0.22 μm membrane. Selection plates were prepared from 90 mL Vogel's agar medium, 10 mL FIGS stock solution, and 400 μL of a 50 mg mL^{-1} hygromycin B stock solution, giving a nominal final hygromycin concentration of 200 μg mL^{-1}. Crossing medium contained 1× crossing salts, 1% sucrose, and 1.8% agar.

S6. Additional Protein Normalization and Image Measurements

For the comparison on day 7 of growth on Avicel, extracellular and intracellular protein measurements were paired for WT, *Δkin-1*, *Ckin-1*, and *Δkin-1*::*kinesin*. The normalized measure for each pair was calculated as (extracellular protein concentration × 50 mL)/(intracellular protein concentration × 1 mL) and expressed as extracellular protein (mg) per mg of intracellular protein. The calculation used the culture and extraction volumes of this experiment. Three paired measurements were available for each strain, and results are presented as the mean ± sample standard deviation. Input concentrations, individual ratios, and calculation records are retained in Supplementary Data

S3. This analysis applies only to the four-strain complementation series on day 7 of growth on Avicel.

Intracellular protein serves as the normalization reference; this measure is not equivalent to dry biomass, viable biomass, protein secretion rate, or a cell lysis assay.

Hyphal branching was measured within the fields shown in Figures 1C and 6C. The actual scale bars in Figures 1C and 6C represent 200 μm and 500 μm, respectively. For each selected main hypha, annotated branching events associated with the main axis and its higher-order branches were counted. The denominator was the length traced along the main-axis centerline, excluding lateral branch lengths. The resulting measure was the number of branches per mm of main hypha. Three main axes or hyphal segments were recorded for each strain in each image set. WT segments with no visible branches were retained as valid zero values. Cases of uncertain branching or connectivity in overlapping hyphal networks remain identified in the annotations in Supplementary Data S3. These measurements are descriptive observations within image fields, not independent biological cultures; no inferential significance tests were applied to them in the supplementary analysis. Symbols in the original figures indicate comparisons against WT using the author-confirmed one-way ANOVA followed by Dunnett's multiple-comparisons test. Figure S8 separately shows hyphal micrographs of independent clones and transformants for qualitative comparison; these images were not included in the axis-segment counts or used for additional significance testing.

The additional RT-qPCR assays used samples grown on Avicel or sucrose for 48 h, with *β-actin* as the reference gene and three independent culture flasks per strain under each condition. Numerical records were summarized descriptively on the original measurement scale using n, the arithmetic mean, and the sample standard deviation. The author confirmed that the statistical symbols in the original figures were derived from one-way ANOVA followed by Dunnett's multiple-comparisons test against WT. Thus, all asterisks and ns denote comparisons against WT under the same condition and at the same time point for the same gene or assay. They do not denote comparisons among engineered strains or direct comparisons against *Δkin-1*.

S7. Sequence Records and Coverage

PCR products amplified from genomic DNA of the final experimental strains were sequenced to examine the P*ccg-1–clr-2* and *T. reesei* kinesin constructs. The available sequence records are provided in Supplementary Data S1 and include annotated reference sequences of 4,021 bp and 9,105 bp, respectively. Four reads align to the P*ccg-1–clr-2* reference sequence and span the promoter–*clr-2* junction. Two reads align to the *T. reesei* kinesin reference sequence, with coverage extending across the boundary between the annotated kinesin region and its downstream flank.

Supplementary Figures

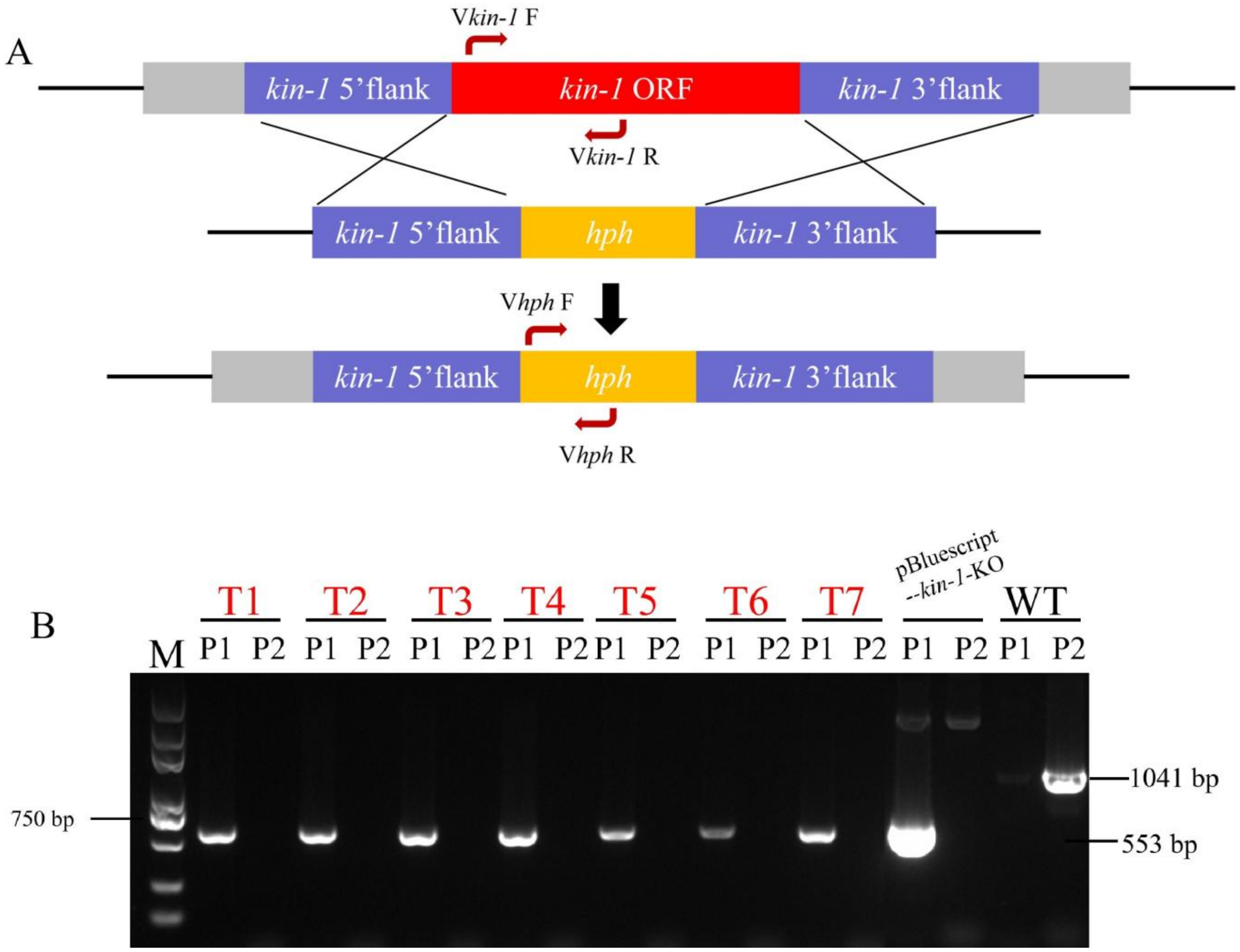

Figure S1. Construction and PCR screening of the *N. crassa Δkin-1* strain. (A) Replacement of the *kin-1* open reading frame with *hph* by homologous recombination. (B) PCR screening of candidates T1–T7. P1: Vhph F/R, with a recorded product of 553 bp; P2: Vkin-1 F/R, with a WT product of 1041 bp. M: DNA size marker; pBluescript-*kin-1*-KO: plasmid control; WT: wild type. Red labels indicate candidates scored as positive in the source figure.

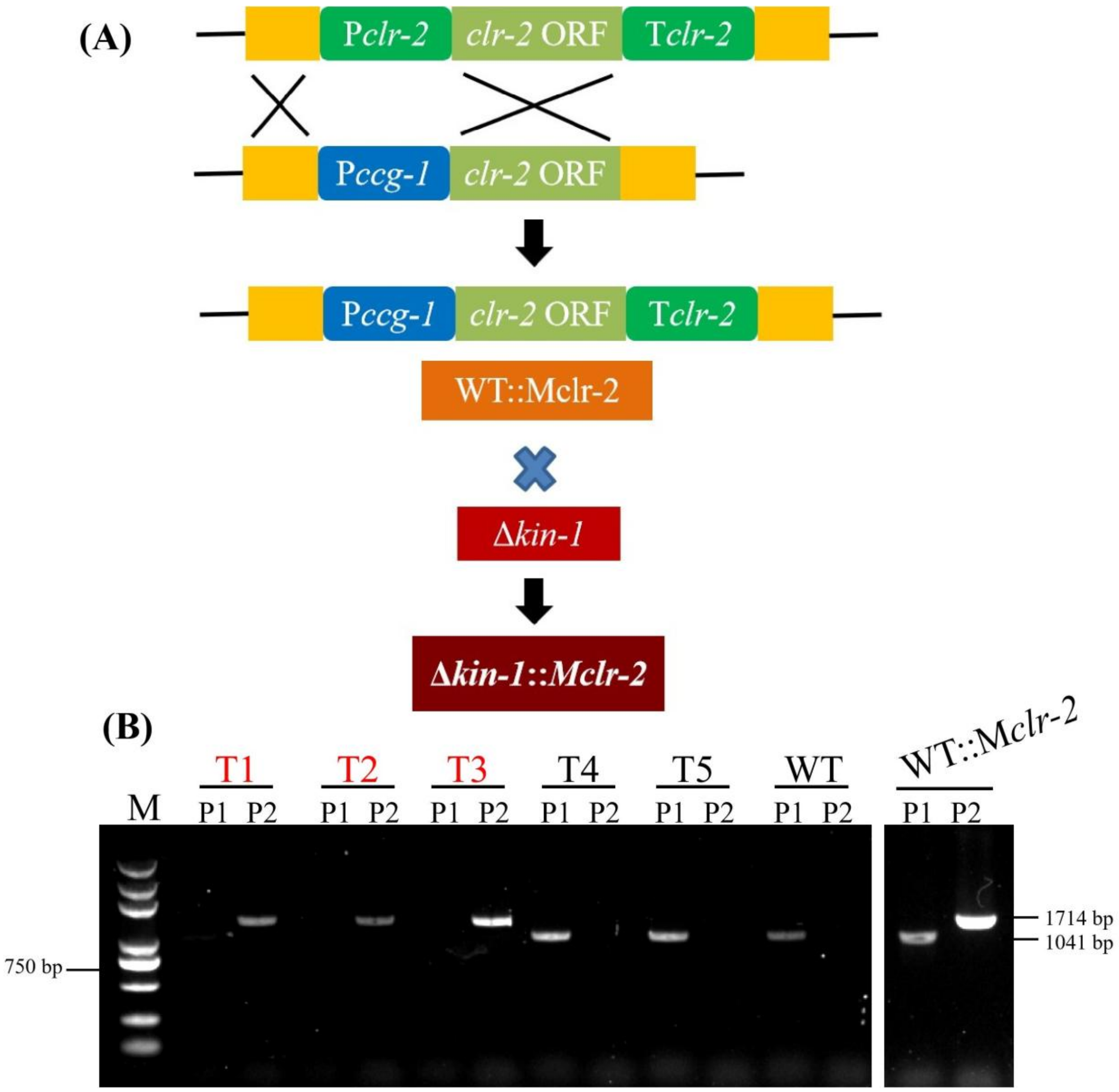

Figure S2. Construction and PCR screening of *Δkin-1*::*Mclr-2*. (A) Schematic of P*ccg-1*-driven *clr-2* expression and crossing with *Δkin-1*. (B) Screening of candidates T1–T5 using WT and WT::*Mclr-2* as controls. P1: Vkin-1 F/R, with a recorded WT product of 1041 bp; P2: VMclr-2 F/R, with a cassette-associated product of 1714 bp. T1–T3 are marked in red as the selected candidates. M: DNA size marker. The expression cassette is integrated at the *his-3* locus; the promoter schematic depicts the cassette structure rather than replacement of the endogenous *clr-2* promoter. The WT::*Mclr-2* control lanes were taken from the lower row of the supplied source photograph, and the candidate and WT lanes from the upper row. White gaps mark the joins between these nonadjacent image regions.

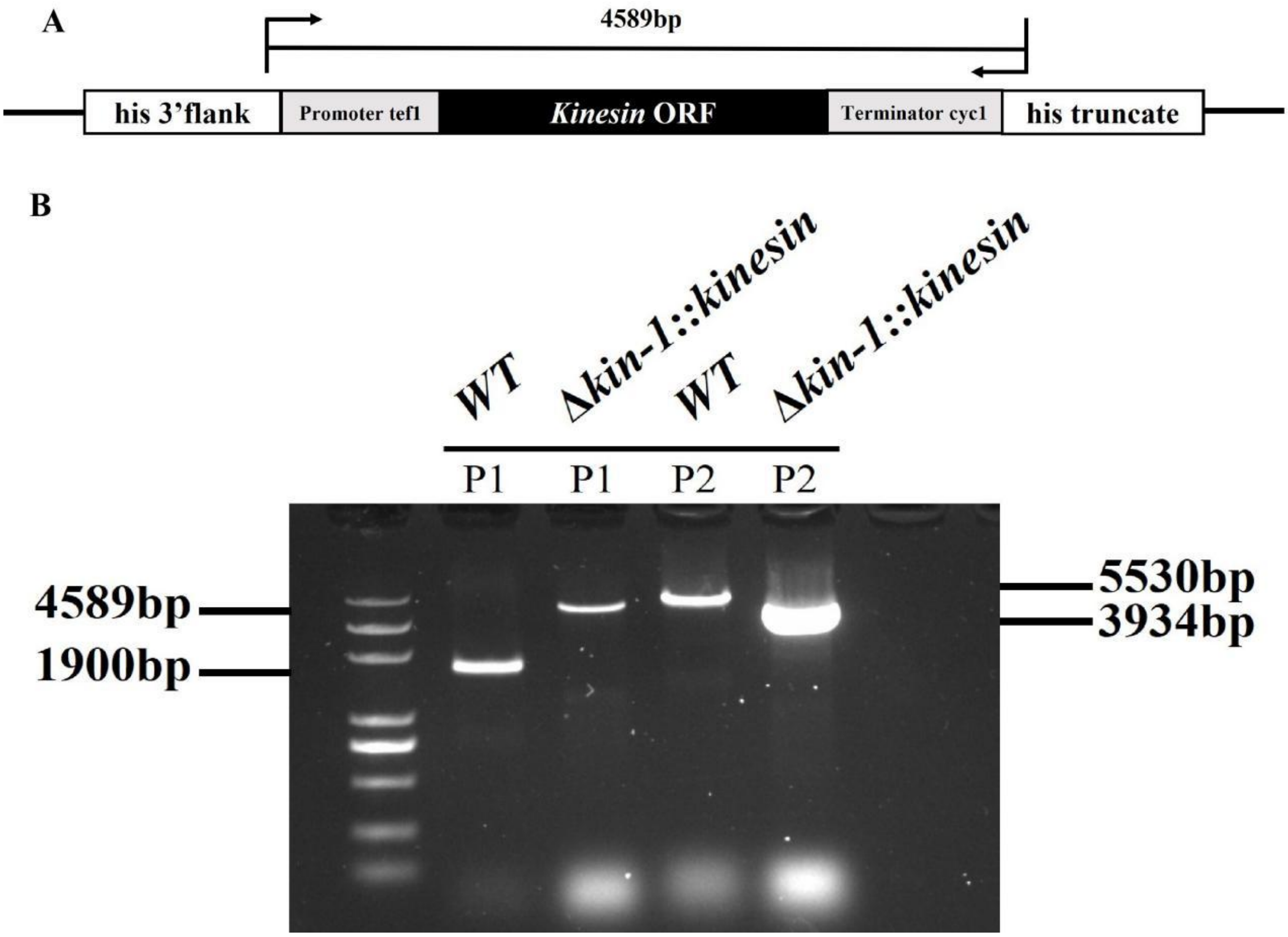

Figure S3. Construction scheme and PCR screening of *Δkin-1*::*kinesin*. (A) The *tef1* promoter, kinesin open reading frame, *cyc1* terminator, and the indicated his locus sequences are labeled. (B) PCR bands of WT and *Δkin-1*::*kinesin*. In the supplied figure, the P1 products of WT and the complemented strain are labeled as 1900 bp and 4589 bp, respectively, and the P2 products as 5530 bp and 3934 bp, respectively.

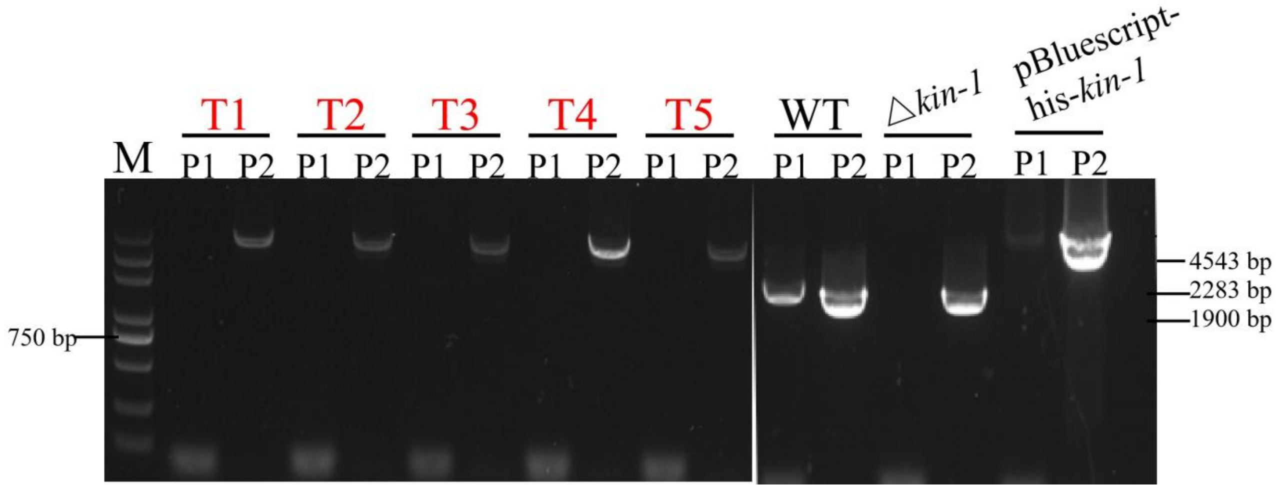

Figure S4. PCR screening of candidate strains for native *kin-1* complementation. M: DNA size marker; T1–T5: candidate *Ckin-1* isolates; WT: wild type; *Δkin-1*: deletion mutant; pBluescript-his-*kin-1*: plasmid control. P1 (Vkin-1 5′ flank F and Vkin-1 R) detects the intact endogenous *kin-1* locus, with an expected product of 2283 bp in WT. P2 (VpLN-Ihis F/R) has an expected complementation-associated product of 4543 bp, whereas the corresponding product in WT and *Δkin-1* is approximately 1900 bp. T1–T5 lacked the target P1 product but yielded the expected P2 product, consistent with the expected complemented genotype. Vertical dividing lines separate the displayed image regions; the source photograph is included in the accompanying original image materials.

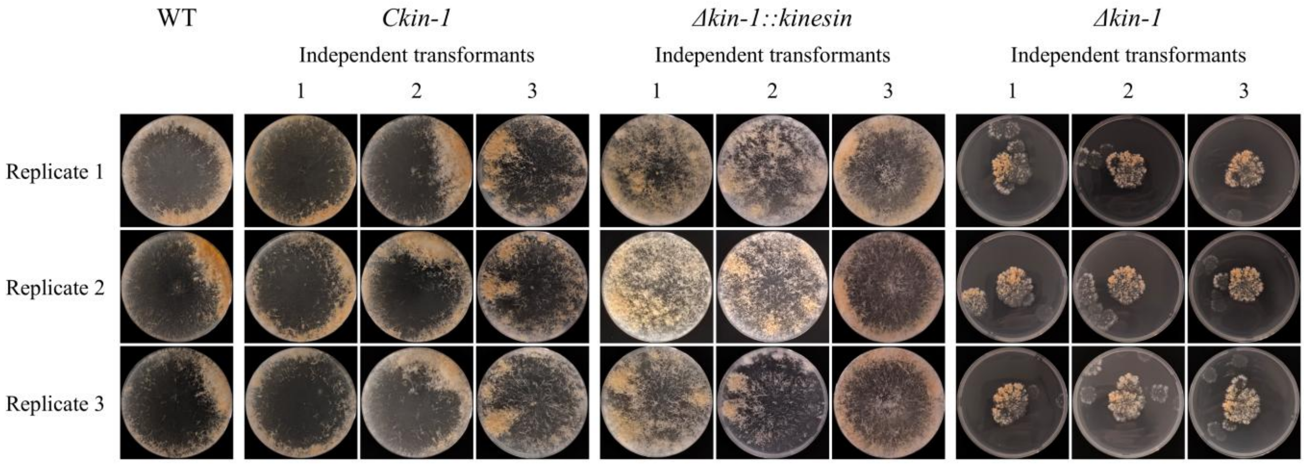

Figure S5. Colony morphology of WT, *Ckin-1*, *Δkin-1*::*kinesin*, and *Δkin-1*. Three replicate plates are shown for WT. For each of the other strains, three independent transformants are shown, with three replicate plates per transformant. Transformants are arranged in columns and replicate plates in rows. Transformant numbers indicate the order of isolates within each strain group. The images are intended for qualitative comparison of colony morphology and do not provide quantitative measurements of conidial abundance or biomass.

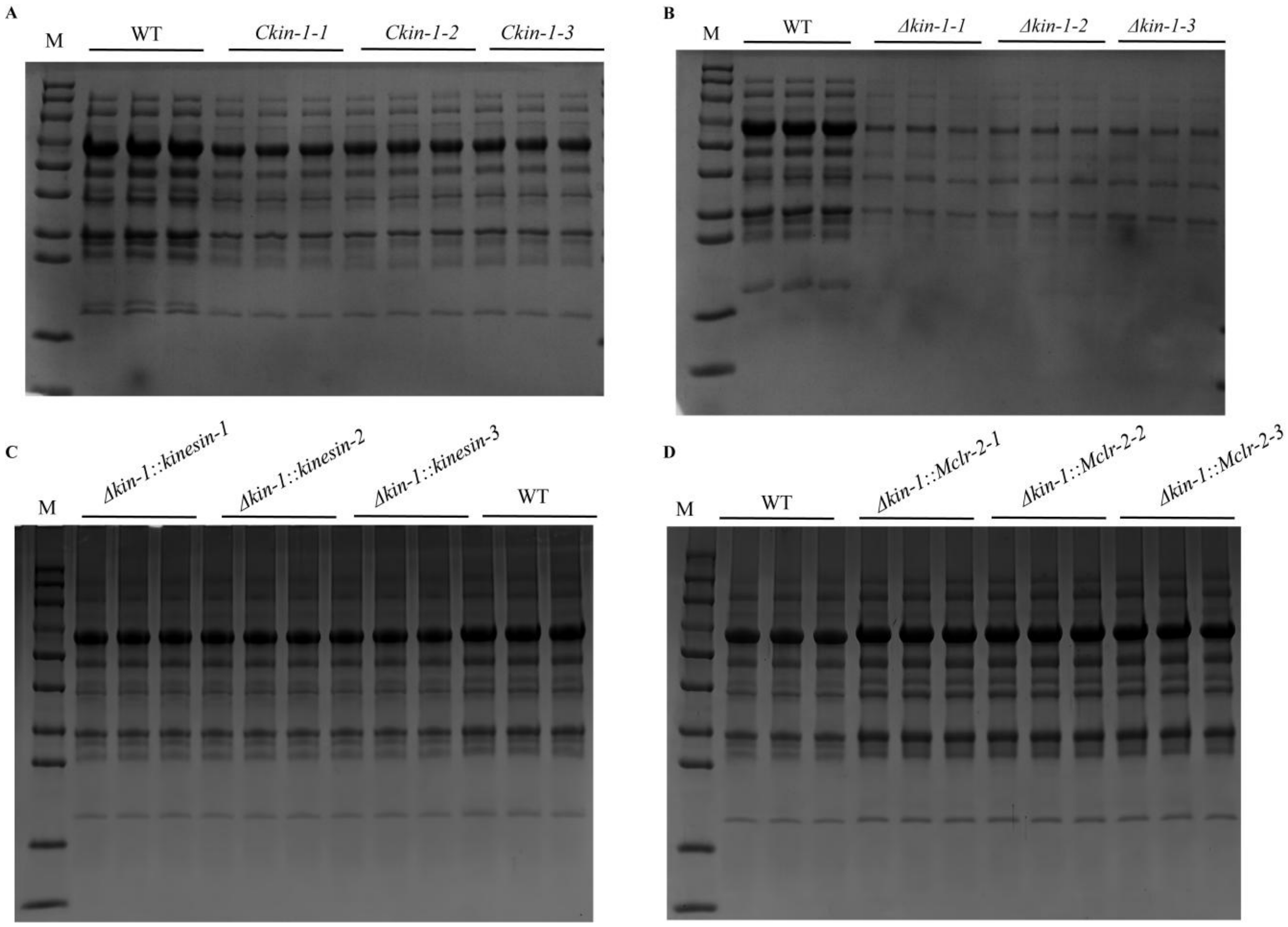

Figure S6. Extracellular protein bands of the indicated isolates. (A) WT and *Ckin-1* isolates 1–3. (B) WT and *Δkin-1* isolates 1–3. (C) *Δkin-1*::*kinesin* isolates 1–3 and WT. (D) WT and *Δkin-1*::*Mclr-2* isolates 1–3. The three lanes under each numbered isolate represent three independent culture flasks. M: protein molecular weight marker. The gels are used for qualitative comparison of extracellular protein banding patterns; they were not used to identify individual bands or infer quantitative differences. Uncropped source gels are provided in Supplementary Data S2. Panel C was flipped horizontally relative to the source gel.

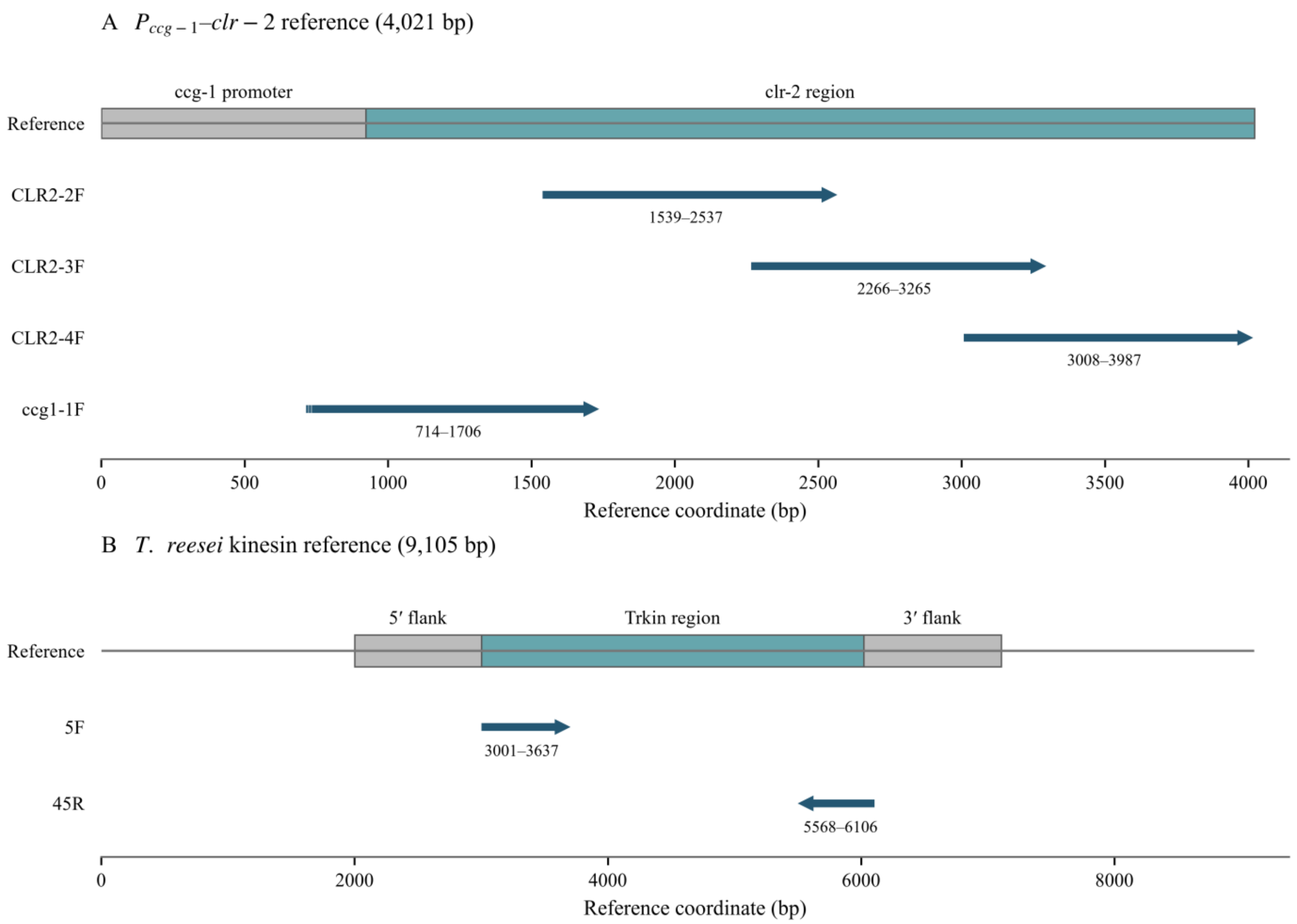

Figure S7. Partial coverage of construct reference sequences by sequencing reads. (A) The P*ccg-1*–*clr-2* reference sequence (4,021 bp), with positions 1–921 annotated as the *ccg-1* promoter and positions 922–4021 as the *clr-2* region. Four sequencing reads cover the indicated intervals, including the promoter–*clr-2* boundary. (B) The *T. reesei* kinesin reference sequence (9,105 bp), with the annotated 5′ flank, Trkin region, and 3′ flank at positions 2001–3000, 3001–6020, and 6021–7105, respectively. Two reads align to positions 3001–3637 and 5568–6106, respectively; the latter spans the boundary between Trkin and the 3′ flank. Reference coordinates are numbered from 1, and intervals include both endpoints. Arrows indicate read orientation; only aligned portions of the reads are shown. These intervals describe the alignments stored in the files and do not represent complete or quality-validated sequence coverage. Smaller alignment gaps and differences between the reference sequences and reads are listed in Supplementary Data S1. Neither panel establishes full-length construct sequence identity, copy number, or validation of the genomic integration site. The PCR products submitted for sequencing were amplified from genomic DNA of the final constructed strains.

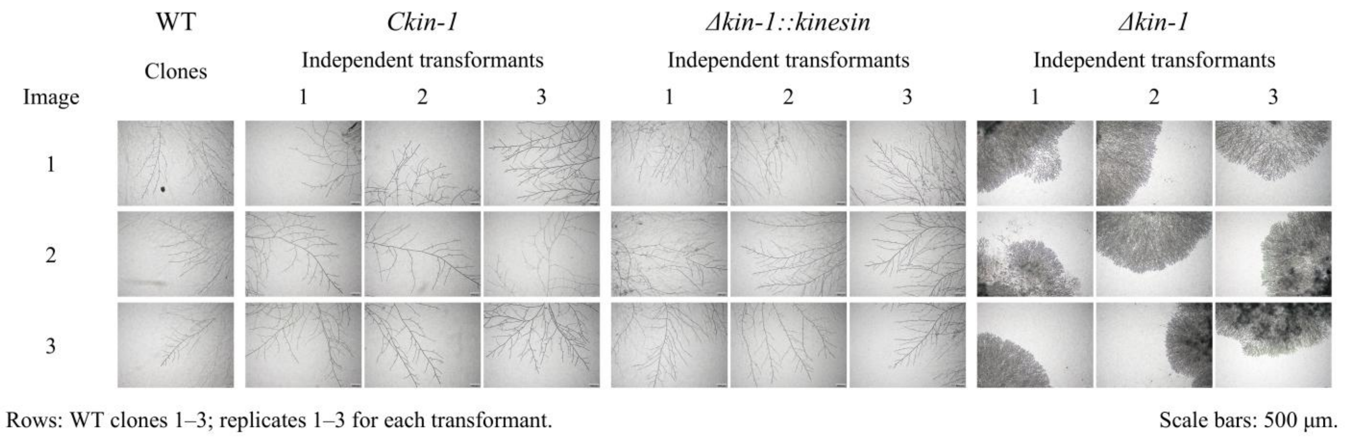

Figure S8. Hyphal morphology of WT, *Ckin-1*, *Δkin-1*::*kinesin*, and *Δkin-1*. The first column shows 3 WT clones from top to bottom. For each of the other strains, 3 independent transformants are arranged in columns; 3 replicate micrographs are shown from top to bottom for each transformant (Image 1–3). Transformant numbers 1–3 indicate the order of isolates within each strain group. Scale bars: 500 μm. The figure is intended for qualitative comparison of hyphal morphology among clones or transformants.

Supplementary Tables

Table S1. Oligonucleotide sequences. Sequences are written in the 5′ to 3′ direction and retained as originally provided.

**Primer**	**Sequence (5′→3′)**
kin-1 5′ flank F	GAGACCGGCAGCGGCCGCCCGAACGGGCATTCAATCTTC
kin-1 5′ flank R	CAATATCATCTTCTGTCTCCGACTATGGCGATTCCCTTGCAG
kin-1 hph F	CTGCAAGGGAATCGCCATAGTCGGAGACAGAAGATGATATTG
kin-1 hph R	GTCTTCTTCACCTCGTTCCCTATTCCTTTGCCCTCGGAC
kin-1 3′ flank F	GTCCGAGGGCAAAGGAATAGGGAACGAGGTGAAGAAGAC
kin-1 3′ flank R	GAGCACTGCGAGGGGCCGATGTTGGGGTTGAGTGTG
BG *kin-1* F	CACACTCAACCCCAACATCGGCCCCTCGCAGTGCTC
BG *kin-1* R	GAAGATTGAATGCCCGTTCGGGCGGCCGCTGCCGGTCTC
Vkin-1 F	ATGTCGTCAAGTGCGAATAG
Vkin-1 R	GATAATGAGTGTTGTTCGAC
Vhph F	GTCGGAGACAGAAGATGATATTG
Vhph R	CTTTGTAGAAACCATCGGCG
kin-1 F	CAACCGTCAAAGATATCGGATATGTCGTCAAGTGCGAATAG
kin-1 R	GAAGGCTTTAATTTGCAAGCTTTACGACTTCTGGAAGAAC
pLN-Ihis F	GTTCTTCCAGAAGTCGTAAAGCTTGCAAATTAAAGCCTTC
pLN-Ihis R	CTATTCGCACTTGACGACATATCCGATATCTTTGACGGTTG
Mate A F	AATCTCGGCAGCACTACCTC
Mate A R	GTGGACGCCGCTGATTTG
Mate a F	ATGGACGGTAACTCGACAC
Mate a R	TCATCACAGTGGGAATGAGAG
Vkin-1 5′ flank F	CAACTCGAACAGCAGGAAAG
VpLN-Ihis-F	TCTGCTAACGTCAAGACACATAGC
VpLN-Ihis-R	GTTAGGTGGGAACGCTTGTATTGC
q actin-F	ACCGCAAGTGCTTCTAAATTCAA
q actin-R	TGCAGAATACACGCGCAATG
q *cbh-1*-F	GGTCCAAGTGAGTACGCGAG
q *cbh-1*-R	CAAACGGGATGTGAGGGGAT
q *clr-2*-F	CTTTTCTGTGGCTGCGAGTG
q *clr-2*-R	TGGTGTGACTCCTTGGAATGT

Primer	Sequence (5′→3′)
VMclr-2 F	GCTTCCAACTTGGTCATCTCG
VMclr-2 R	GCTTGGACAGTCTCGATATGG

Table S2. Phenotypic changes following native complementation, *clr-2* expression, and heterologous complementation. Descriptions apply only to the carbon sources and measures examined; a nonsignificant difference from WT does not establish equivalence. "Not measured" indicates that the comparison was not performed in this study.

**Measure and condition**	**Native complementation, *Ckin-1***	***Δkin-1*::*Mclr-2***	***Δkin-1*::*kinesin***
Growth and colony morphology on sucrose	Colony appearance and extension were closer to WT; no equivalence test was performed.	This comparison was not performed.	Colony appearance and radial growth were closer to WT; no equivalence test was performed.
Image-based branching measurements on sucrose	The descriptive branching measure was lower than in *Δkin-1* in the fields shown.	Not measured.	The descriptive branching measure was lower than in *Δkin-1* in the fields shown.
Extracellular protein concentration on Avicel	Higher than in *Δkin-1* but still below WT late in the culture period.	Concentrations were higher than in *Δkin-1* and WT late in the culture period.	Higher than in *Δkin-1* but still below WT late in the culture period.
Protein ratio on day 7 of growth on Avicel	Restored to near-WT levels; the mean was 3.750 mg/mg, compared with 2.272 for *Δkin-1*. Descriptive comparison only.	Not measured.	Restored to near-WT levels; the mean was 4.227 mg/mg, compared with 2.272 for *Δkin-1*. Descriptive comparison only.
clr-2 and *cbh-1* transcript levels after 48 h on Avicel	Not measured.	Mean levels of both transcripts were higher than in *Δkin-1*; these observations do not establish statistical significance for direct comparisons against *Δkin-1* or between different *Mclr-2* genetic backgrounds.	Not measured.
clr-2 and *cbh-1* transcript levels after 48 h on sucrose	Not measured.	Mean levels of both transcripts were higher than in WT and *Δkin-1*; symbols in the original figures indicate comparisons against WT only.	Not measured.
Enzyme activities on Avicel	In the updated records, mean pNPC activity was 0.10315 IU/mL, above WT; mean pNPG activity was 0.01293 IU/mL, below WT but above *Δkin-1*. References used for blank subtraction in FPA and CMCase calculations remain to be checked, so the extent of restoration is not assessed for these two activities. Descriptive comparisons only; see Table S4.	Mean values of all four enzyme activities were higher than in *Δkin-1*. Symbols in the original figures indicate comparisons against WT only; no statistical significance is assigned to direct differences from *Δkin-1* or among engineered strains.	In the updated records, mean pNPC and pNPG activities were 0.01666 and 0.01687 IU/mL, respectively, both below WT but above *Δkin-1*. References used for blank subtraction in FPA and CMCase calculations remain to be checked, so no overall conclusion about restoration is drawn. Descriptive comparisons only; see Table S4.

Measure and condition	Native complementation, *Ckin-1*	*Δkin-1*::*Mclr-2*	*Δkin-1*::*kinesin*
Extracellular protein and enzyme activities on sucrose	Not measured.	Protein concentration was highest late in the culture period; mean values of all four enzyme activities were higher than in *Δkin-1* and WT::*Mclr-2*. These comparisons of means do not establish statistical significance for direct comparisons.	Not measured.
Hydrolysis of straw with enzymes from Avicel cultures at a protein loading of 0.1 mg	Not measured.	Mean sugar yields from *Miscanthus* and rice straw were higher than those of WT/*Δkin-1*; sugar yield from corn straw was lower than that of WT.	Not measured.
Hydrolysis of straw with enzymes from sucrose cultures at a protein loading of 0.1 mg	Not measured.	Mean sugar yields were highest for all three straw substrates tested.	Not measured.

Image-based branching measurements are descriptive observations within images. Table S2 distinguishes observed changes in means from inferential conclusions. The author confirmed that all asterisks and ns in the original figures indicate comparisons against WT using one-way ANOVA followed by Dunnett's multiple-comparisons test. These symbols do not demonstrate significant restoration relative to *Δkin-1* or significant differences among engineered strains. In this revision, the enzyme activity descriptions for the complementation series and Table S4 were updated using "酶活性数据.xlsx". Figure 7D remains unchanged and has not been redrawn from the updated table; its bar heights, error bars, and asterisks should not be interpreted as statistical outputs from the updated table.

Table S3. Descriptive numerical summaries of the normalized protein, additional qPCR, and *Mclr-2* enzyme activity datasets. n denotes the number of supplied values; the mean and SD are the arithmetic mean and sample standard deviation on the original measurement scale. The protein ratio is expressed as extracellular protein (mg) per mg of intracellular protein, qPCR values are relative expression levels, and enzyme activities are expressed in IU/mL. No additional statistical tests, p values, or significance symbols are introduced in this table. The author confirmed that the annotations in the original figures were based on one-way ANOVA followed by Dunnett's multiple-comparisons test against WT under the same condition and at the same time point for the same gene or assay. These comparisons do not test restoration directly relative to *Δkin-1* or differences among engineered strains.

Dataset	Strain	n	Mean	SD
Protein ratio on day 7 of growth on Avicel	WT	3	3.91786	1.31816
	Δkin-1	3	2.27165	0.174314
	Ckin-1	3	3.7504	0.962912
	Δkin-1::*kinesin*	3	4.22723	0.463818

Dataset	Strain	n	Mean	SD
cbh-1 on Avicel	WT	3	1.04746	0.102737
	Δkin-1	3	0.361111	0.0633727
	WT::*Mclr-2*	3	1.43234	0.0845615
	Δkin-1::*Mclr-2*	3	1.41511	0.193866
clr-2 on Avicel	WT	3	0.999151	0.0979705
	Δkin-1	3	0.224533	0.0850763
	WT::*Mclr-2*	3	1.50021	0.132061
	Δkin-1::*Mclr-2*	3	1.30247	0.0790172
cbh-1 on sucrose	WT	3	0.954606	0.0913158
	Δkin-1	3	0.823634	0.0547717
	WT::*Mclr-2*	3	10.9162	0.818889
	Δkin-1::*Mclr-2*	3	11.1837	1.02514
clr-2 on sucrose	WT	3	0.955743	0.127709
	Δkin-1	3	0.807066	0.0761609
	WT::*Mclr-2*	3	11.0738	0.848336
	Δkin-1::*Mclr-2*	3	11.4476	0.786241
FPA on Avicel	WT	3	0.135075	0.020681
	Δkin-1	3	0.0396718	0.00570724
	WT::*Mclr-2*	3	0.532702	0.0612346
	Δkin-1::*Mclr-2*	3	0.564977	0.0177252
FPA on sucrose	WT	3	0.00375678	0.000727351
	Δkin-1	3	0.0013673	0.000876393
	WT::*Mclr-2*	3	0.220839	0.0126431
	Δkin-1::*Mclr-2*	3	0.304261	0.0351678
CMCase on sucrose	WT	3	0.0310083	0.00305798
	Δkin-1	3	0.0427243	0.00598994
	WT::*Mclr-2*	3	0.738125	0.0464283
	Δkin-1::*Mclr-2*	3	1.51078	0.0482537
CMCase on Avicel	WT	3	0.517653	0.0221821
	Δkin-1	3	0.147774	0.00582623
	WT::*Mclr-2*	3	1.4457	0.0640171
	Δkin-1::*Mclr-2*	3	1.57144	0.08228

Dataset	Strain	n	Mean	SD
pNPC hydrolytic activity on sucrose	WT	3	0.0256114	0.000269232
	Δkin-1	3	0.0340946	0.00200722
	WT::*Mclr-2*	3	0.0811528	0.00713344
	Δkin-1::*Mclr-2*	3	0.190879	0.00638549
pNPC hydrolytic activity on Avicel	WT	3	0.0452035	0.00878754
	Δkin-1	3	0.0207222	0.000326198
	WT::*Mclr-2*	3	0.135321	0.00481483
	Δkin-1::*Mclr-2*	3	0.160615	0.0148459
pNPG hydrolytic activity on sucrose	WT	3	0.757984	0.0784691
	Δkin-1	3	0.0110905	0.000442762
	WT::*Mclr-2*	3	2.22979	0.113569
	Δkin-1::*Mclr-2*	3	7.29042	0.0420071
pNPG hydrolytic activity on Avicel	WT	3	4.1579	0.0632284
	Δkin-1	3	0.0158265	0.000644971
	WT::*Mclr-2*	3	7.76001	0.234475
	Δkin-1::*Mclr-2*	3	8.68871	0.275958

In the additional qPCR experiments, $n = 3$ independent cultures per strain under each condition were sampled at 48 h, with *β-actin* as the reference gene. In the day 7 protein-ratio dataset, $n = 3$ per strain denotes three paired measurements. The enzyme activity summaries in Table S3 retain the three values supplied for each group in the original *Mclr-2* enzyme activity dataset; these values are not reclassified as independently constructed transformants. The latest enzyme activity source data for the complementation series are presented separately in Table S4 and are not pooled with the *Mclr-2* data.

Table S4. Updated enzyme activity records for the complementation series on day 7 of growth on Avicel. Activities are expressed in IU/mL, and SD denotes the sample standard deviation. The number of records denotes only the number of numerical values used for descriptive calculations in this table, not a verified number of independent culture replicates.

Assay	Strain or source-table label	Number of records	Mean (IU/mL)	SD（IU/mL）
FPA	WT	3	0.0676619	0.00470672
FPA	*Δkin-1*	3	0.015564	0.000431308
FPA	*Ckin-1*	3	0.0345214	0.000127769
FPA	*Δkin-1*::*kinesin*	3	0.169296	0.218286
CMCase	WT	3	0.116821	0.0898006
CMCase	*Δkin-1*	3	0.0356012	0.00146327
CMCase	*Ckin-1*	3	0.331768	0.431034
CMCase	*Δkin-1*::*kinesin*	3	0.100943	0.00592435
pNPC	WT	3	0.0274255	0.00404993
pNPC	*Δkin-1*	3	0.00241817	0.000634853
pNPC	*Ckin-1*	3	0.103154	0.00360516
pNPC	*Δkin-1*::*kinesin*	3	0.0166599	0.000374952
pNPG	WT	3	0.0990844	0.0132016
pNPG	*Δkin-1*	3	0.00162123	3.19938e-05
pNPG	*Ckin-1*	3	0.0129283	0.000889985
pNPG	*Δkin-1*::*kinesin*	3	0.0168728	6.802e-05

Supplementary Data

Supplementary Data S1. Annotated reference sequences, original sequence records, embedded sequencing reads and chromatograms, alignment coordinates, and differences between the reference sequences and reads for the P*ccg-1–clr-2* and *T. reesei* kinesin constructs.

Supplementary Data S2. Uncropped gels corresponding to Figures 7A and S6, together with the supplied gel image of the CLR-2 fragment submitted for sequencing and a table linking the displayed images to their sources.

Supplementary Data S3. Numerical data, image-based branching records and annotations, day 7 extracellular/intracellular protein calculations, qPCR relative expression datasets, protein-normalized straw hydrolysis data, and descriptive summaries of n, means, and SDs. The files retain the supplied measurements and normalization formulas without introducing separately chosen tests of all pairwise group comparisons or exact p values.

Supplementary Data S4. An unmodified copy of the author’s further-updated “酶活性数据.xlsx”, containing 48 final values and the existing formulas for four enzyme activities in the complementation series on day 7. The original file has not been altered; matters requiring verification, including references to empty cells and replicate records, are described in the notes to Table S4.
